# Supplementary material for: Mix and match: Patchwork domain evolution of the land plant-specific Ca2+-permeable mechanosensitive channel MCA
Source: PLoS One. 2021 Apr 15;16(4):e0249735. doi: 10.1371/journal.pone.0249735 (PMC8049495; doi:10.1371/journal.pone.0249735)

S1 Appendix. Proposed domain structure of MCA. (*a*) For the MCA protein, the ARPK domain, at the N-terminus, and PLAC8, at the C-terminus, was proposed previously, and it was shown that MCA has an EF hand-like and a coiled-coil region [7]. (*b*) The biological function of MCA proteins was tested. As a result, the MCA functional domain at the N-terminus, which was previously described as ARPK domain and part of the EF hand-like region, was proposed [18]. This domain was analyzed in the present study and has been registered as MCA^{func} domain in the Pfam database

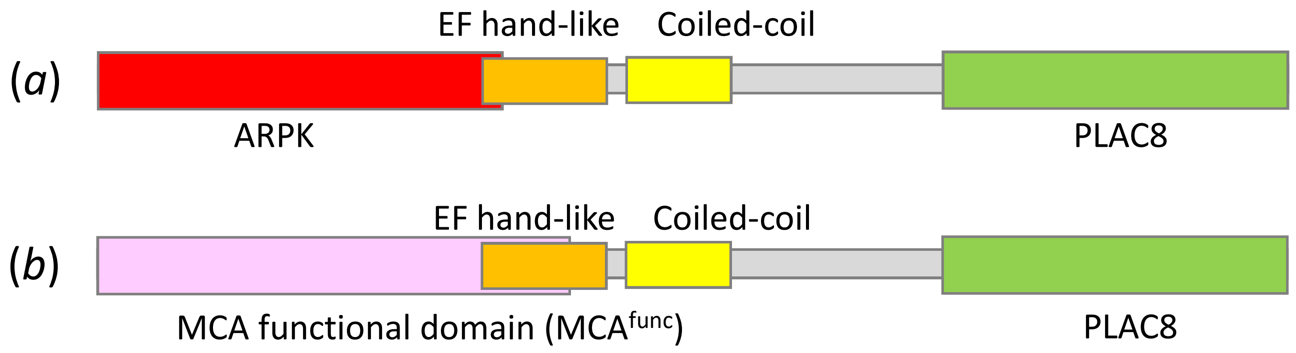

Supplement: S1 Appendix — (a) For the MCA protein, the ARPK domain, at the N-terminus, and PLAC8, at the C-terminus, was proposed previously, and it was shown that MCA has an EF hand-like and a coiled-coil region [7]. (b) The biological function of MCA proteins was tested. As a result, the MCA functional domain at the N-terminus, which was previously described as ARPK domain and part of the EF hand-like region, was proposed [18]. This domain was analyzed in the present study and has been registered as MCAfunc domain in the Pfam database. (PDF) [file pone.0249735.s001.pdf]
