# Supplementary material for: Mix and match: Patchwork domain evolution of the land plant-specific Ca2+-permeable mechanosensitive channel MCA
Source: PLoS One. 2021 Apr 15;16(4):e0249735. doi: 10.1371/journal.pone.0249735 (PMC8049495; doi:10.1371/journal.pone.0249735)
Supplement: S2 Appendix — (PDF) [file pone.0249735.s002.pdf]

S2 Appendix. List of proteomes used in this study. BUSCO-C: % of complete genes. * Gymnosperm proteomes obtained from the plaza database (https://bioinformatics.psb.ugent.be/plaza/) were analyzed in this study.

	ID	Taxon	Vernacular name	Order	Database	Proteome ID	No of proteins	BUSCO-C	BUSCO lineage
1	CHLRE	Chlamydomonas reinhardtii	chlorophytes	Chlamydomonadales	UniProt	UP000006906	18829	98.8%	chlorophyta_odb10
2	VOLCA	Volvox carteri f. nagariensis	chlorophytes	Chlamydomonadales	UniProt	UP000001058	14335	96.6%	chlorophyta_odb10
3	KLENI	Klebsormidium nitens	charophytes	Klebsormidiales	UniProt	UP000054558	16251	96.9%	viridiplantae_odb10
4	CHABU	Chara braunii	charophytes	Charales	UniProt	UP000265515	35576	72.2%	viridiplantae_odb10
5	MARPO	Marchantia polymorpha	hornworts	Marchantiales	UniProt	UP000244005	21856	96.7%	viridiplantae_odb10
6	MapoRu	Marchantia polymorpha subsp. ruderalis	hornworts	Marchantiales	Uniprot	UP000077202	17951	91.3%	viridiplantae_odb10
7	PHYPA	Physcomitrella patens	bryophytes	Funariales	UniProt	UP000006727	30858	97.6%	viridiplantae_odb10
8	SELML	Selaginella moellendorffii	lycophytes	Selaginellales	UniProt	UP000001514	33150	89.4%	viridiplantae_odb10
9	CMI	Cycas micholitzii	gymnosperm	Cycadales	plaza*	na	28901	36.3%	viridiplantae_odb10
10	TBA	Taxus baccata	gymnosperm	Cupressales	plaza*	na	32062	60.7%	viridiplantae_odb10
11	AMBTC	Amborella trichopoda	angiosperm	Amborellales	UniProt	UP000017836	27371	95.3%	viridiplantae_odb10
12	MUSAM	Musa acuminata subsp. malaccensis	angiosperm	Zingiberales	UniProt	UP000012960	36474	91.3%	viridiplantae_odb10
13	ORYSJ	Oryza sativa subsp. Japonica	angiosperm	Poales	UniProt	UP000059680	48903	83.8%	viridiplantae_odb10
14	MAIZE	Zea mays	angiosperm	Poales	Uniprot	UP000007305	99253	96.2%	viridiplantae_odb10
15	SORBI	Sorghum bicolor	angiosperm	Poales	UniProt	UP000000768	41380	99.5%	viridiplantae_odb10
16	AQUCA	Aquilegia coerulea	angiosperm	Ranunculales	UniProt	UP000230069	37018	99.1%	viridiplantae_odb10
17	VITVI	Vitis vinifera	angiosperm	Vitales	Uniprot	UP000009183	29907	96.2%	viridiplantae_odb10
18	POPTR	Populus trichocarpa	angiosperm	Malpighiales	UniProt	UP000006729	53336	98.4%	viridiplantae_odb10
19	MEDTR	Medicago truncatula	angiosperm	Fabales	UniProt	UP000002051	57066	97.2%	viridiplantae_odb10
20	CUCSA	Cucumis sativus	angiosperm	Cucubitales	Uniprot	UP000029981	23744	96.2%	viridiplantae_odb10
21	GOSRA	Gossypium raimondii	angiosperm	Malvales	Uniprot	UP000032304	66534	99.1%	viridiplantae_odb10
22	BRAOL	Brassica oleracea var. oleracea	angiosperm	Brassicales	Uniprot	UP000032141	58535	93.6%	viridiplantae_odb10
23	ARATH	Arabidopsis thaliana	angiosperm	Brassicales	UniProt	UP000006548	39346	99.8%	viridiplantae_odb10
24	ERYGU	Erythranthe guttata	angiosperm	Lamiales	UniProt	UP000030748	28677	95.1%	viridiplantae_odb10
25	SOLLC	Solanum lycopersicum	angiosperm	Solanales	Uniprot	UP000004994	34650	93.6%	viridiplantae_odb10
