# Supplementary material for: Mix and match: Patchwork domain evolution of the land plant-specific Ca2+-permeable mechanosensitive channel MCA
Source: PLoS One. 2021 Apr 15;16(4):e0249735. doi: 10.1371/journal.pone.0249735 (PMC8049495; doi:10.1371/journal.pone.0249735)
Supplement: S3 Appendix — (PDF) [file pone.0249735.s003.pdf]

S3 **Appendix. List of genome and transcriptome databases interrogated in this study, and the list of MCA genes included in the MCA phylogenetic analyses.**

														MCA HMMsearch				MCAfunc HMMsearch				PLAC8 HMMsearch			
vernacular	clade	clade	clade	order	family	Taxon	database	note	gene ID	chromosome/Genome scaffolds	ID in matrix	ID in proteome analyses	length (AA)	HMM E value	from	to	length	HMM E value	from	to	length	HMM E value	from	to	length
Hornworts	-	-	-	Anthocerotales	Anthocerotaceae	Anthoceros angustus	NCBI Genome	GCA_010909165.1	MU008170.1	scaffold 21	HW.MU008170.ANTAN		789	2.50E-148	3	406	404	1.70E-75	1<br>139	141<br>167	141<br>29	9.10E-14	1	99	99
Liverworts	-	-	-	Marchantiales	Marchantiaceae	Marchantia polymorpha	Phytozome v.12		Mapoly0134s0009	scaffold_134	LW.0134s0009.MARPO	A0A2R6W7G4_MARPO	483	3.30E-155	4	421	418	3.90E-65	5	166	162	8.70E-19	1	99	99
Bryophytes	-	-	-	Funariales	Funariaceae	Physcomitrella patens	Phytozome v.12	v3.3	Pp3c12_21520V3	Chromosome 12	MO.12_21520V3.PHYPA	A0A2K1JRK9_PHYPA	438	3.50E-172	4	421	418	5.80E-75	5	167	163	1.00E-17	1	98	98
									Pp3c8_990V3	Chromosome 08	MO.8_990V3.PHYPA	A0A2K1K5M7_PHYPA	445	4.80E-170	5	421	417	1.50E-72	7	166	160	1.30E-16	1	96	96
Bryophytes	-	-	-	Sphagnales	Sphagnaceae	Sphagnum fallax	Phytozome v.12	v0.5	Sphfalx0076s0017	super_76	MO.0076s0017.SPHFA		440	1.30E-164	4	420	417	2.50E-75	4	167	164	2.40E-17	1	98	98
Lycophytes	-	-	-	Selaginellales	Selaginellaceae	Selaginella moellendorffii	Phytozome v.12	v1.0	Sm_93428	scaffold_14	LY.93428.SELML	D8S521/D8RGG8	396	1.10E-163	8	406	399	5.40E-79	8	167	160	4.10E-21	1	99	99
Monilophytes	-	-	-	Salviniales	Salviniaceae	Azolla filiculoides	FernBase		Azfi_s0316.g064349	scaffold_0316	FN.s0316.g064349.AZOFI		600	6.20E-148	3	376	374	8.30E-78	4	166	163	9.30E-12	1	80	80
Monilophytes	-	-	-	Salviniales	Salviniaceae	Salvinia cucullata	FernBase		Sacu_v1.1_s0057.g014785	scaffold_0057	FN.s0057.g014785.SALCU		771	2.60E-169	4	421	418	2.00E-78	4	167	164	9.10E-20	1	99	99
Gymnosperms	-	-	-	Ginkgoales	Ginkgoaceae	Ginkgo biloba	1KP		SGTW_scaffold_2002341	unknown	GY.2002341.GBI		422	6.10E-173	1	421	421	2.30E-76	1<br>83	63<br>167	63<br>85	7.10E-16	1	99	99
Gymnosperms	-	-	-	Cycadales	Cycadaceae	Cycas micholitzii	1KP		XZUY_scaffold_2011951	unknown	GY._2011951.CMI	CMI00009383	434	1.10E-202	1	421	421	1.90E-95	1	167	167	7.60E-17	1	99	99
Gymnosperms	-	-	-	Welwitschiales	Welwitschiaceae	Welwitschia mirabilis	1KP		TOXE_scaffold_2008256	unknown	GY._2008256.WMI		376	1.50E-168	1	402	402	2.20E-88	2	167	166	2.5E-16	1	99	99
Gymnosperms	-	-	-	Cupressales	Taxaceae	Taxus baccata	PLAZA	TBA	TBA00013714	unknown	GY._00013714.TBA	TBA00013714	419	2.70E-195	3	421	419	5.80E-95	4	167	164	5.90E-17	1	99	99
Angiosperms	-	-	-	Amborellales	Amborellaceae	Amborella trichopoda	Phytozome v.12	v1.0	evm_27.model.AmTr_v1.0_scaffold00152.28	scaffold_00152	AG.Amtr_00152.28.AMBTC	W1PMV8_AMBTC	422	9.60E-231	1	422	422	1.30E-103	1	167	167	3.60E-18	1	99	99
Angiosperms	-	-	-	Nymphaeales	Nymphaeaceae	Nymphaea thermarum	NCBI Genome	GCA_011799795.1	JAANDH010000004.1	scaffold4	AG.JAANDH010000004.1.NYMTH		140	3.80E-165	1	407	407	1.80E-99	1	166	166	3.10E-06	1	99	99
Angiosperms	-	magnoliids	-	Magnoliales	Magnoliaceae	Magnolia grandiflora	1KP		WBOD_scaffold_2018894	unknown	AG._2018894.MAGGR		446	1.20E-237	1	422	422	6.50E-105	1	167	167	6.40E-18	1	99	99
									WBOD_scaffold_2019688	unknown	AG._2019688.MAGGR		290	2.90E-106	7<br>285	242<br>319	236<br>35	7.40E-70	8	166	159	no hits			
Angiosperms	-	magnoliids	-	Laurales	Lauraceae	Cinnamomum camphora	1KP		BCGB_scaffold_2010188	unknown	AG._2010188.CINCA		413	1.60E-215	1	422	422	9.20E-85	1	167	167	7.00E-18	1	99	99
									BCGB_scaffold_2008909	unknown	AG._2008909.CINCA		438	1.80E-203	1	420	420	1.40E-103	1	167	167	8.40E-14	19	99	81
									BCGB_scaffold_2048332	unknown	AG._2048332.CINCA		308	1.40E-105	14<br>123<br>296	53<br>251<br>420	40<br>129<br>125	2.00E-24	14<br>123	53<br>167	40<br>45	8.60E-17	1	99	99
Monocots	-	-	-	Alismatales	Araceae	Spirodela polyrhiza	Phytozome v.12	v2.0	Spipo1G0068000	pseudo1	AGM.1G0068000.SPIPO		401	5.30E-209	1	421	421	4.50E-104	1	167	167	4.80E-17	1	99	99
									Spipo17G0028800	pseudo17	AGM.17G0028800.SPIPO		362	9.60E-180	1<br>336	262<br>421	262<br>86	7.90E-101	1	167	167	4.90E-12	50	99	50
									Spipo11G0041200	pseudo11	AGM.11G0041200.SPIPO		416	5.00E-212	4	422	419	4.50E-103	4	167	164	1.50E-17	1	99	99
									Spipo0G0150600	pseudo0	AGM.0G0150600.SPIPO		312	2.90E-122	103	422	320	2.20E-32	103	165	63	2.30E-16	1	99	99
Monocots	-	-	-	Alismatales	Zosteraceae	Zostera marina	Phytozome v.12	v2.2	Zosma1g03120.1	scaffold 1	AGM.1g03120.1.ZOSMR		403	1.70E-203	3	421	419	7.10E-100	3	166	164	3.70E-18	1	99	99
Monocots	-	-	-	Asparagales	Asparagaceae	Asparagus officinalis	KEGG genome	T0543	aof:109847658	unknown	AGM.aof_109847658.ASPOF		419	5.90E-228	2	422	421	9.60E-102	1	167	167	4.50E-19	1	99	99
									aof:109837680	unknown	AGM.aof_109837680.ASPOF		414	5.30E-223	7	420	414	8.60E-102	7	167	161	2.90E-17	1	99	99
Monocots	-	commelinids	-	Poales		Ananas comosus	Phytozome v.12	v3	Aco010382.1	LG03	AGM.Aco010382.1.ANACO		420	3.00E-237	2	422	421	7.40E-106	1	167	167	2.40E-17	1	99	99
Monocots	-	commelinids	-	Poales	Poaceae	Oryza sativa	Phytozome v.12		Os03g0157300	chromosome 3	AGM.Os03g0157300.ORYSJ	Q10RJ5_ORYSJ	418	2.90E-229	2	422	421	5.30E-101	1	167	167	1.60E-19	1	99	99
Monocots	-	commelinids	-	Poales	Poaceae	Panicum hallii	Phytozome v.12	v2.0	Pahal.I00468.1	chromosome 9	AGM.Pahal.I00468.1.PANHA		419	9.20E-224	2	422	421	9.60E-101	1	167	167	5.80E-18	1	99	99
Monocots	-	commelinids	-	Poales	Poaceae	Setaria viridis	Phytozome v.12	v1.1	Sevir.9G539400.1	chromosome 9	AGM.9G539400.1.SETVI		419	2.80E-223	2	422	421	7.00E-101	1	167	167	3.70E-18	1	99	99
Monocots	-	commelinids	-	Poales	Poaceae	Sorghum bicolor	Phytozome v.12		Sobic.001G499200.1	chromosome 1	AGM.001G499200.1.SORBI	C5WXT4_SORBI	421	1.70E-225	2	422	421	5.80E-101	1	167	167	3.20E-19	1	99	99
Monocots	-	commelinids	-	Poales	Poaceae	Brachypodium distachyon	Phytozome v.12	v3.1	Bradi1g74650.2	Bd1	AGM.1g74650.2.BRADI		413	3.40E-223	7	422	416	1.00E-96	7	167	161	1.10E-18	1	99	99
Monocots	-	commelinids	-	Zingiberales	Musaceae	Musa acuminata	Phytozome v.12	v1	GSMUA_Achr7T03850_001	chromosome 7	AGM.7T03850_001.MUSAC	M0TEH5_MUSAM	416	1.20E-218	2	421	420	1.40E-102	1	167	167	2.50E-18	1	99	99
									GSMUA_Achr1T26450_001	chromosome 1	AGM.1T26450_001.MUSAC	M0S3A2_MUSAM	419	6.50E-224	2	421	420	6.20E-101	1	167	167	7.70E-19	1	99	99
Eudicots	-	-	-	Ranunculales	Ranunculaceae	Aquilegia coerulea	Phytozome v.12	v3.1	Aqcoe6G217900.1	chromosome 6	AGE.6G217900.1.AQUCO	A0A2G5C987_AQUCA	422	1.30E-233	1	422	422	5.30E-106	1	167	167	3.60E-18	1	99	99
Eudicots	-	-	-	Proteales	Nelumbonaceae	Nelumbo nucifera	KEGG genome	T04131	nnu_104603638	unknown	AGE._104603638.NELNU		420	3.00E-241	1	421	421	6.40E-104	1	167	167	1.10E-17	1	99	99
									nnu_104604268	unknown	AGE._104604268.NELNU		409	2.40E-234	1	421	421	6.20E-104	1	167	167	6.10E-19	1	99	99
Eudicots	core eudicots	superrosids	-	Saxifragales	Crassulaceae	Kalanchoe fedtschenkoi	Phytozome v.12	v1.0	Kaladp0008s0106.1	scaffold 8	AGE.0008s0106.1.KALFE		419	2.40E-217	2	421	420	1.10E-103	1	167	167	2.30E-18	1	99	99
									Kaladp0067s0162.1	scaffold 67	AGE.0067s0162.1.KALFE		418	5.60E-227	2	422	421	1.40E-103	1	167	167	3.10E-16	1	99	99
Eudicots	core eudicots	superrosids	rosids	Vitales	Vitaceae	Vitis vinifera	Phytozome v.12	v2.1	VIT_200s0349g00020.3_GSVIVT0	unknown	AGE.200s0349g00020.3.VITVI	F6H5I8_VITVI	319	5.60E-173	103	422	320	2.00E-105	1	167	167	3.30E-17	1	99	99
Eudicots	core eudicots	superrosids	rosids	Malpighiales	Linaceae	Linum usitatissimum	Phytozome v.12	v1.0	Lus10025955	scaffold_319	AGR._10025955.LINUS		419	8.40E-229	2	422	421	1.30E-102	1	167	167	6.30E-18	1	99	99
									Lus10041875	scaffold_272	AGR._10041875.LINUS		477	2.00E-225	3	422	420	2.00E-102	3	167	165	2.30E-17	1	99	99
									Lus10028421	scaffold_413	AGR._10028421.LINUS		526	2.50E-226	3	422	420	2.40E-102	3	167	165	2.30E-18	1	99	99
									Lus10014256	scaffold_275	AGR._10014256.LINUS		430	1.10E-229	2	422	421	1.10E-101	1	167	167	2.40E-18	1	99	99
Eudicots	core eudicots	superrosids	rosids	Malpighiales	Euphorbiaceae	Manihot esculenta	Phytozome v.12	v6.1	Manes.02G108200.1	chromosome 2	AGR.02G108200.MANES		419	2.90E-238	3	422	420	1.70E-107	3	167	165	7.20E-18	1	99	99
									Manes.01G149200.1	chromosome 1	AGR.01G149200.MANES		419	5.30E-238	3	422	420	1.20E-106	3	167	165	1.10E-17	1	99	99
									Manes.07G134400.1	chromosome 7	AGR.07G134400.MANES		388	4.30E-148	11<br>295	240<br>420	230<br>126	4.20E-64	1<br>186	160<br>223	160<br>38	1.50E-17	1	99	99
									Manes.07G064800.1	chromosome 7	AGR.07G064800.MANES		300	5.10E-57	10	136	127	1.10E-52	10	137	128	no hits	na	na	na
Eudicots	core eudicots	superrosids	rosids	Malpighiales	Euphorbiaceae	Ricinus communis	Phytozome	v0.1	28962.m000451	scaffold 28962	AGR.28962.m000451.RICCO		283	1.70E-151	3	276	274	1.80E-107	3	167	165	no hits	na	na	na
									29729.m002275	scaffold 29729	AGR.29729.m002275.RICCO		393	3.90E-144	11	421	411	5.70E-64	11	167	157	6.60E-17	1	99	99
Eudicots	core eudicots	superrosids	rosids	Malpighiales	Salicaceae	Populus trichocarpa	Phytozome v.12	v3.0	Potri.005G110000.1	chromosome 5	AGR.005G110000.1.POPTR	U5GEX3_POPTR	461	4.80E-231	2	417	416	9.10E-105	1	167	167	9.50E-17	1	99	99
									Potri.010G184700.1	chromosome 10	AGR.010G184700.1.POPTR	A0A2K1YWA1_POPTR	390	3.50E-143	11	420	410	4.10E-63	11	167	157	8.80E-18	1	99	99
Eudicots	core eudicots	superrosids	rosids	Malpighiales	Salicaceae	Salix purpurea	Phytozome v.12	v1.0	SapurV1A.0130s0180.1	chromosome 5	AGR.0130s0180.1.SALPU		457	2.30E-225	2	409	408	3.40E-101	1	167	167	3.00E-17	1	99	99
									SapurV1A.0061s0640.1	chromosome 10	AGR.0061s0640.1.SALPU		388	3.00E-143	11<br>295	236<br>420	226<br>126	3.10E-64	11	167	157	1.90E-17	1	99	99
Eudicots	core eudicots	superrosids	rosids	Fabales	Fabaceae	Medicago truncatula	Phytozome v.12	Mt4.0v1	Medtr8g075150.1	chromosome 8	AGR.8g075150.1.MEDTR	G7LB65_MEDTR	424	2.70E-222	1	422	422	4.10E-104	1	167	167	4.90E-16	1	99	99
									Medtr5g022670.4	chromosome 5	AGR.5g022670.4.MEDTR	G7JKX8_MEDTR	416	6.80E-228	2	422	421	9.60E-104	1	167	167	1.10E-17	1	99	99
Eudicots	core eudicots	superrosids	rosids	Fabales	Fabaceae	Trifolium pratense	Phytozome v.12	v2	Tp57577_TGAC_v2_mRNA20153	Tp57577_TGAC_v2_LG4	AGR.mRNA20153.TRIPR		425	1.10E-226	1	422	422	4.50E-104	1	167	167	3.60E-15	1	99	99
									Tp57577_TGAC_v2_mRNA10902	Tp57577_TGAC_v2_LG3	AGR.mRNA10902.TRIPR		417	3.20E-229	1	422	422	8.20E-105	1	167	167	7.80E-18	1	99	99
Eudicots	core eudicots	superrosids	rosids	Fabales	Fabaceae	Lotus japonicus	KEGG genome	T04348	Lj2g3v1670960	chromosome 2	AGR.2g3v1670960.LOTJA		416	6.80E-230	2	422	421	2.40E-103	1	167	167	1.10E-18	1	99	99
									Lj0g3v0324739	unknown	AGR.0g3v0324739.LOTJA		413	8.90E-225	1	421	421	9.70E-107	1	167	167	1.90E-17	1	99	99
Eudicots	core eudicots	superrosids	rosids	Rosales	Rosaceae	Prunus persica	Phytozome v.12	v2.1	Prupe.7G172600.1	Pp07	AGR.7G172600.1.PRUPE		416	2.90E-240	1	421	421	1.10E-107	1	167	167	5.50E-18	1	99	99
									Prupe.2G255300.1	Pp02	AGR.2G255300.1.PRUPE		422	5.10E-122	16<br>300	239<br>421	224<br>122	5.90E-52	7	167	161	5.30E-19	1	99	99
Eudicots	core eudicots	superrosids	rosids	Cucurbitales	Cucurbitaceae	Cucumis sativus	Phytozome v.12	v1.0	Cucsa.322780.1	scaffold03080	AGR.322780.1.CUCSA	A0A0A0L3N0_CUCSA	418	1.60E-233	3<br>294	421<br>402	419<br>109	1.30E-104	3	167	165	7.30E-18	1	99	99
									Cucsa.043820.1	scaffold00542	AGR.043820.1.CUCSA	A0A0A0KGR0_CUCSA	372	9.30E-191	3	260	258	1.30E-100	3	167	165	2.90E-19	1	99	99
Eudicots	core eudicots	superrosids	rosids	Myrtales	Myrtaceae	Eucalyptus grandis	Phytozome v.12	v2.0	Eucgr.I02323.1	chromosome 9	AGR.I02323.1.EUCGR		472	1.30E-216	2	397	396	1.80E-103	1	166	166	4.90E-08	1	75	75
									Eucgr.J02474.1	chromosome 10	AGR.J02474.1.EUCGR		348	3.80E-171	76	419	344	1.70E-52	76	167	92	1.40E-15	1	99	99

vernacular	clade	clade	clade	order	family	Taxon	database	note	gene ID	chromosome/Genome scaffolds	ID in matrix	ID in proteome analyses	length (AA)	HMM E value	from	to	*length*	HMM E value	from	to	*length*	HMM E value	from	to	*length*
Eudicots	core eudicots	superrosids	rosids	Sapindales	Rutaceae	Citrus sinensis	Phytozome v.12	v1.1	orange1.1g014734m	scaffold00017	AGR.1g014734m.CITSI		419	**3.90E-236**	2	422	*421*	**9.00E-105**	1	167	*167*	**5.70E-17**	1	99	*99*
									orange1.1g020555m	scaffold00021	AGR.1g020555m.CITSI		329	**2.10E-104**	14	239	*226*	**1.10E-61**	14	167	*154*	**no hits**	na	na	*na*
															291	340	*50*								
Eudicots	core eudicots	superrosids	rosids	Malvales	Malvaceae	Gossypium raimondii	Phytozome v.12	v2.1	Gorai.003G007400.1	chromosome 3	AGR.003G007400.1.GOSRA	A0A0D2MHL8_GOSRA	418	**2.10E-242**	2	422	*421*	**6.20E-105**	1	167	*167*	**1.00E-17**	1	99	*99*
									Gorai.007G192000.1	chromosome 7	AGR.007G192000.1.GOSRA	A0A0D2THW9_GOSRA	416	**3.50E-239**	1	422	*422*	**2.30E-105**	1	167	*167*	**2.80E-17**	1	99	*99*
Eudicots	core eudicots	superrosids	rosids	Brassicales	Brassicaceae	Arabidopsis thaliana	Phytozome v.12	TAIR10	AT4G35920.1 (MCA1)	chromosome 4	AGR.AtMCA1.ARATH	MCAC1_ARATH	419	**3.10E-224**	1	422	*422*	**5.40E-104**	1	167	*167*	**2.90E-20**	1	99	*99*
									AT2G17780.1 (MCA2)	chromosome 2	AGR.AtMCA2.ARATH	MCAC2_ARATH	417	**1.10E-191**	1	421	*421*	**8.70E-95**	1	167	*167*	**2.30E-17**	1	99	*99*
Eudicots	core eudicots	superrosids	rosids	Brassicales	Brassicaceae	Brassica oleracea capitata	Phytozome v.12	v1.0	Bol018686	chromosome 7	AGR.Bol018686.BRAOC		421	**4.70E-219**	1	422	*422*	**1.40E-102**	1	167	*167*	**8.00E-18**	1	99	*99*
									Bol029039	chromosome 1	AGR.Bol029039.BRAOC		419	**4.40E-223**	1	422	*422*	**4.70E-105**	1	167	*167*	**5.20E-18**	1	99	*99*
Eudicots	core eudicots	superrosids	rosids	Brassicales	Brassicaceae	Brassica oleracea oleracea	KEGG genome	T04124	A0A0D3A1N3_BRAOL; boe_1063(	chromosome 1	AGR.boe_106303054.BRAOL	A0A0D3A1N3_BRAOL	420	**1.40E-224**	1	422	*422*	**2.00E-104**	1	167	*167*	**6.10E-20**	1	99	*99*
									A0A0D3DHX0_BRAOL;boe_1063(	chromosome 1	AGR.boe_106301973.BRAOL	A0A0D3DHX0_BRAOL	412	**2.20E-221**	1	422	*422*	**1.00E-102**	1	167	*167*	**9.10E-20**	1	99	*99*
									A0A0D3E374_BRAOL;boe_10631	chromosome 9	AGR.boe_106313387.BRAOL	A0A0D3E374_BRAOL	411	**5.70E-190**	1	242	*242*	**5.70E-101**	1	167	*167*	**4.80E-20**	1	99	*99*
															293	406	*114*								
Eudicots	core eudicots	superrosids	rosids	Brassicales	Brassicaceae	Capsella rubella	Phytozome v.12	v1.0	Carubv10004901m	scaffold_7 5'end F	AGR.10004901m.CAPRU		421	**9.20E-221**	1	422	*422*	**5.50E-104**	1	167	*167*	**3.60E-18**	1	99	*99*
									Carubv10013809m	scaffold_3 3'end R	AGR.10013809m.CAPRU		417	**1.30E-201**	1	421	*421*	**4.90E-99**	1	166	*166*	**9.70E-18**	1	99	*99*
Eudicots	core eudicots	superrosids	rosids	Brassicales	Caricaeae	Carica papaya	Phytozome v.12	v0.4	evm.model.supercontig_6.294	supercontig_6	AGR.6.294.CARPA		420	**1.90E-239**	1	421	*421*	**8.90E-109**	1	167	*167*	**7.20E-18**	1	99	*99*
									evm.model.supercontig_19.136	supercontif_19	AGR.19.136.CARPA		345	**1.80E-104**	11	235	*225*	**4.80E-63**	11	167	*157*	**3.70E-04**	1	37	*37*
															295	341	*47*								
Eudicots	core eudicots	superasterids	-	Caryophyllales	Amaranthaceae	Amaranthus hypochondriac	Phytozome v.12	v1.0	AHYPO_016156-RA	scaffold 297	AGA._016156.AMAHP		378	**3.90E-185**	6	421	*416*	**3.70E-87**	6	139	*134*	**3.10E-15**	1	99	*99*
																			155	167					
									AHYPO_016414-RA	scaffold 310	AGA._016414.AMAHP		375	**1.50E-186**	2	339	*338*	**1.40E-100**	6	167	*162*	**7.80E-09**	1	36	*36*
															373	421	*49*						75	99	*25*
									AHYPO_007217-RA	scaffold 56	AGA._007217.AMAHP		385	**1.00E-175**	3	314	*312*	**8.00E-93**	3	167	*165*	**3.20E-09**	2	99	*98*
															338	421	*84*								
Eudicots	core eudicots	superasterids	-	Caryophyllales	Chenopodiaceae	Beta vulgaris	EnsemblPlants	RefBeet-1.2.2	KMT09333	chromosome 6	AGA.KMT09333.BETVU		407	**3.00E-217**	9	422	*414*	**2.1E-99**	9	167	*159*	**4.1E-19**	1	99	*99*
									KMS99790	chromosome 1	AGA.KMS99790.BETVU		406	**1.80E-190**	3	421	*419*	**1.7E-90**	3	167	*165*	**5.4E-21**	1	99	*99*
									KMT09333b	chromosome 6	AGA.KMT09333b.BETVU		407	**3.00E-217**	9	422	*414*	**2.1E-99**	9	167	*159*	**4.1E-19**	1	99	*99*
									KMS99790b	chromosome 1	AGA.KMS99790b.BETVU		406	**1.80E-190**	3	421	*419*	**1.7E-90**	3	167	*165*	**5.4E-21**	1	99	*99*
Eudicots	core eudicots	superasterids	asterids	Solanales	Solanaceae	Nicotiana tabacum	KEGG Genome	T04643	LOC107799659_NtMCA2	unknown	AGA.NtMCA2.TOBAC		419	**3.60E-232**	2	422	*421*	**1.70E-104**	1	167	*167*	**3.30E-18**	1	99	*99*
									LOC107804487_NtMCA1	unknown	AGA.NtMCA1.TOBAC		419	**3.60E-232**	2	422	*421*	**1.70E-102**	1	167	*167*	**5.60E-19**	1	99	*99*
																							60	76	*17*
									LOC107766330	unknown	AGA.107766330.TOBAC		419	**2.40E-233**	2	422	*421*	**8.20E-103**	1	167	*167*	**5.60E-19**	1	99	*99*
Eudicots	core eudicots	superasterids	asterids	Solanales	Solanaceae	Solanum lycopersicum	Phytozome v.12	iTAG2.4	Solyc02g083540.2.1	SL2.50ch02	AGA.02g083540.2.1.SOLLC	A0A3Q7F6L0_SOLLC	418	**2.10E-228**	4	422	*419*	**5.60E-102**	1	167	*167*	**3.60E-18**	1	99	*99*
									Solyc03g095820.2.1 has two doma	SL2.50ch03	AGA.03g095820.2.1.SOLLC	A0A3Q7FPG3_SOLLC	390	**1.80E-136**	13	237	*225*	**1.30E-56**	13	167	*155*	**1.60E-18**	1	99	*99*
															288	420	*133*								
Eudicots	core eudicots	superasterids	asterids	Solanales	Solanaceae	Solanum tuberosum	Phytozome v.12	v4.03	PGSC0003DMT400009236	ST4.03ch02	AGA.DMT400009236.SOLTU		418	**9.70E-228**	4	422	*419*	**8.20E-102**	1	167	*167*	**4.30E-18**	1	99	*99*
									PGSC0003DMT400058105	ST4.03ch03	AGA.DMT400058105.SOLTU		432	**1.70E-227**	2	407	*406*	**5.80E-102**	1	167	*167*	**8.70E-18**	1	99	*99*
									PGSC0003DMT400053095	ST4.03ch03	AGA.DMT400053095.SOLTU		389	**1.70E-141**	13	231	*219*	**2.60E-62**	13	167	*155*	**2.30E-18**	1	99	*99*
															289	420	*132*								
Eudicots	core eudicots	superasterids	asterids	Lamiales	Phrymaceae	Erythranthe guttata	Phytozome v.12	v2.0	Migut.N03178.1	scaffold 14	AGA.N03178.1.ERYGU	A0A022RAP2_ERYGU	389	**1.60E-186**	4	421	*418*	**2.30E-95**	3	164	*162*	**5.90E-19**	1	99	*99*
									Migut.H02182.1	scaffold 8	AGA.H02182.1.ERYGU	A0A022PTL3_ERYGU	413	**6.90E-217**	6	421	*416*	**6.70E-100**	6	167	*162*	**8.90E-17**	1	99	*99*
									Migut.C00882.1	scaffold 3	AGA.C00882.1.ERYGU	A0A022QT97_ERYGU	404	**1.10E-195**	2	421	*420*	**5.30E-94**	1	165	*165*	**1.00E-16**	1	99	*99*
Eudicots	core eudicots	superasterids	asterids	Asterales	Asteraceae	Helianthus annuus	EnsemblPlants	HanXRQr1.0	OTF93336	chromosome 16	AGA.OTF93336.HELEN		413	**1.80E-231**	1	422	*422*	**5.80E-105**	1	167	*167*	**5.20E-19**	1	99	*99*
									OTF96330	chromosome 15	AGA.OTF96330.HELEN		420	**7.80E-236**	1	422	*422*	**9.90E-104**	1	167	*167*	**4.10E-19**	1	99	*99*
									OTF98085	chromosome 14	AGA.OTF98085.HELEN		428	**2.10E-214**	1	421	*421*	**8.70E-103**	1	166	*166*	**1.50E-16**	1	99	*99*
									OTG15114	chromosome 9	AGA.OTG15114.HELEN		544	**2.80E-197**	1	398	*398*	**2.70E-99**	1	167	*167*	**6.10E-06**	1	47	*47*
Eudicots	core eudicots	superasterids	asterids	Apiales	Apiaceae	Daucus carota	Phytozome v.12	v2.0	DCAR_011609	chromosome 3	AGA.011609.DAUCA		478	**1.40E-220**	1	407	*407*	**3.60E-104**	1	167	*167*	**4.20E-15**	1	99	*99*
									DCAR_014040	chromosome 4	AGA.014040.DAUCA		315	**2.10E-156**	2	315	*314*	**4.60E-100**	1	167	*167*	**no hits**	na	na	*na*
