# Supplementary figures and images for: Mix and match: Patchwork domain evolution of the land plant-specific Ca2+-permeable mechanosensitive channel MCA
Source: PLoS One. 2021 Apr 15;16(4):e0249735. doi: 10.1371/journal.pone.0249735 (PMC8049495; doi:10.1371/journal.pone.0249735)

**S4 Appendix.** Profile HMM logo of the full MCA protein, PTHR46604:SF3

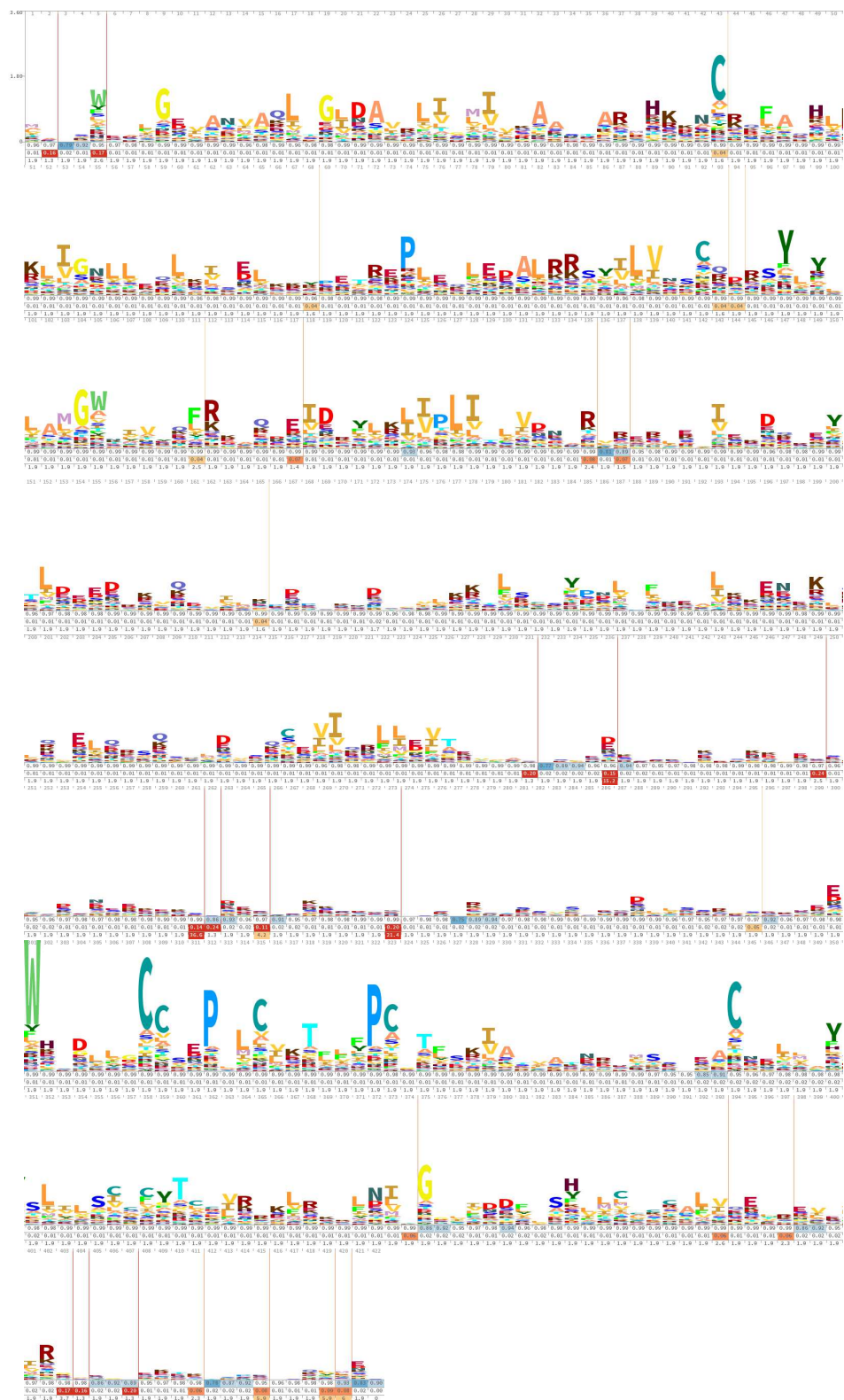

Supplement: S4 Appendix — (PDF) [file pone.0249735.s004.pdf]

(a)

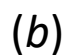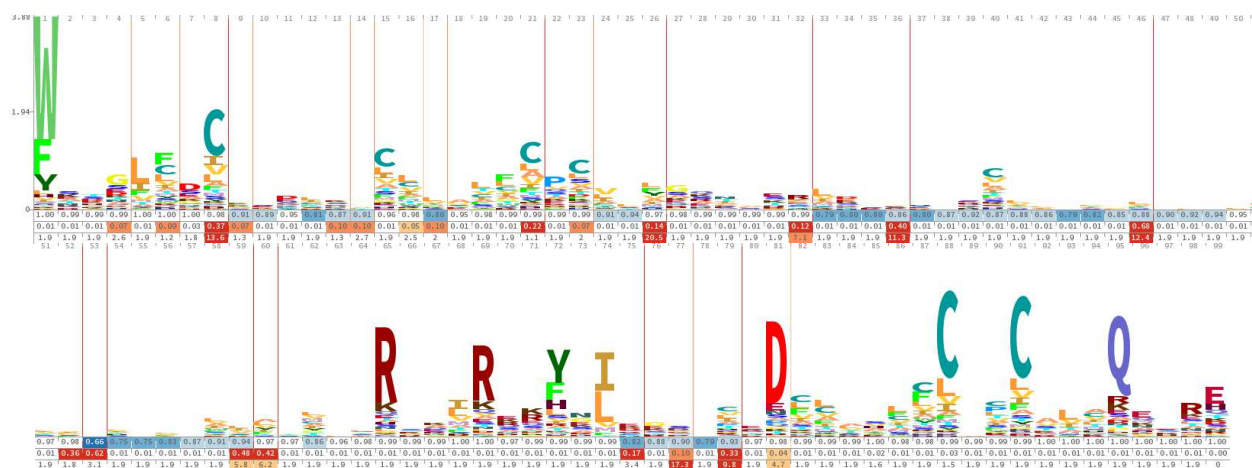

Supplement: S5 Appendix — (a) MCAfunc.hmm generated in this study, (b) PLAC8.hmm (PF04749). (PDF) [file pone.0249735.s005.pdf]

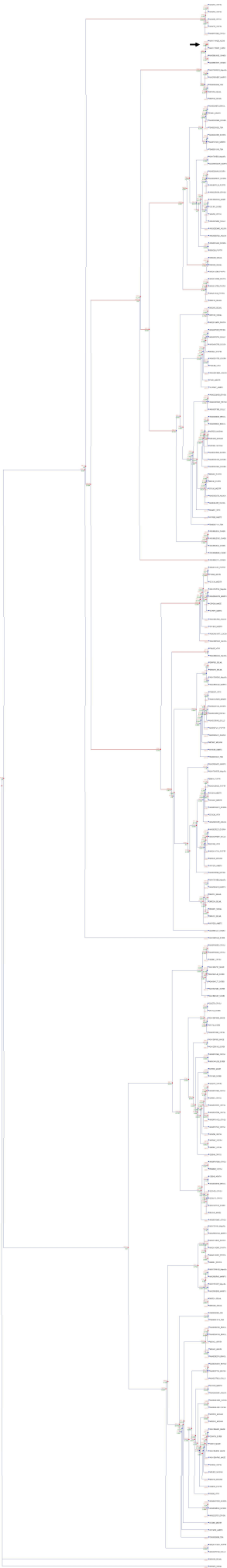

Supplement: S10 Appendix — The possible root position is marked in red (arrow). (PDF) [file pone.0249735.s010.pdf]

**S13 Appendix. PLAC8 domain ML tree. (I) MCA clade**  
**(II) Clade with proteins retaining DUF2985 + PLAC8**

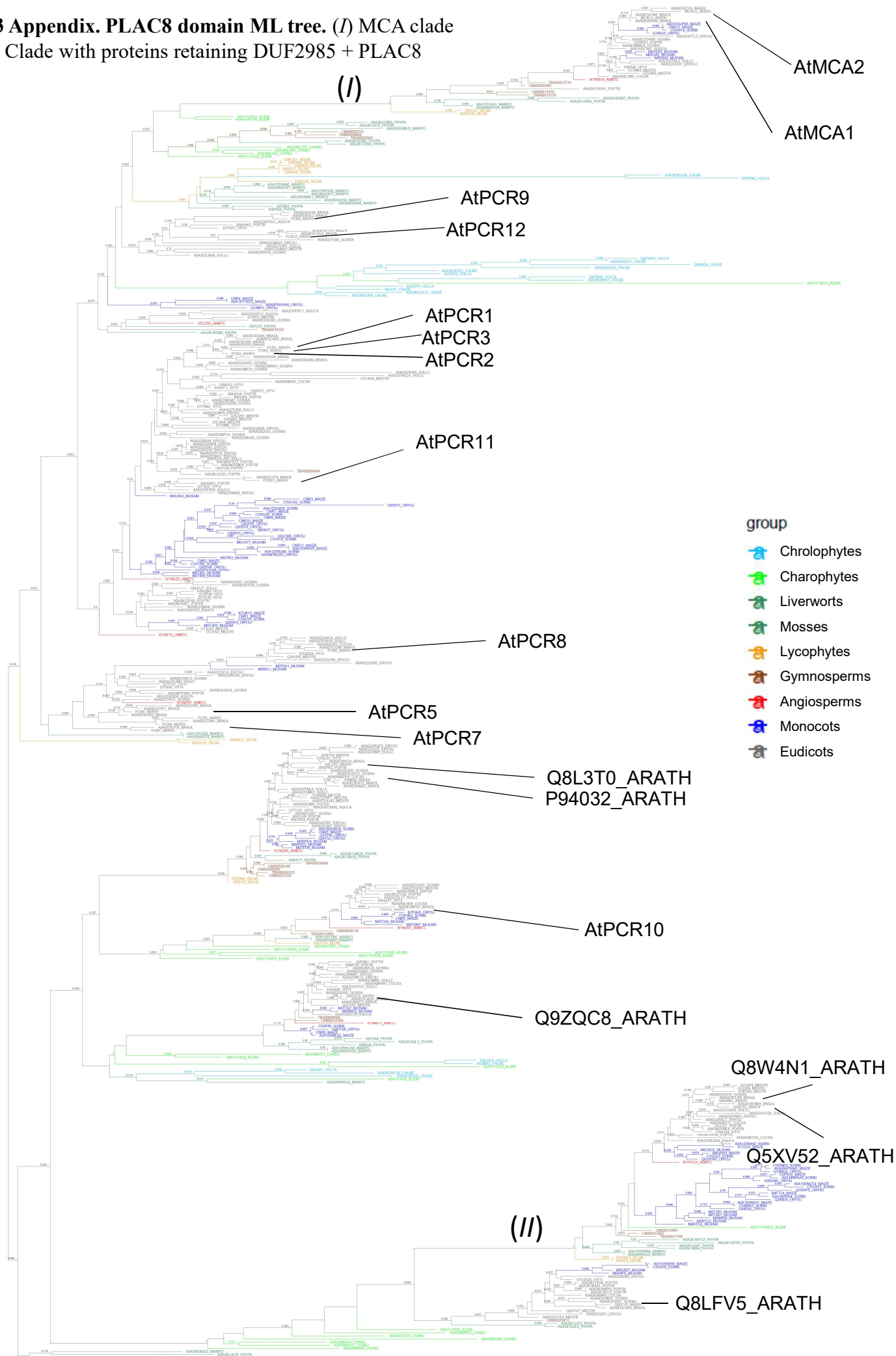

Supplement: S13 Appendix — (I) MCA clade (II) Clade with proteins retaining DUF2985 + PLAC8. (PDF) [file pone.0249735.s013.pdf]

(a)

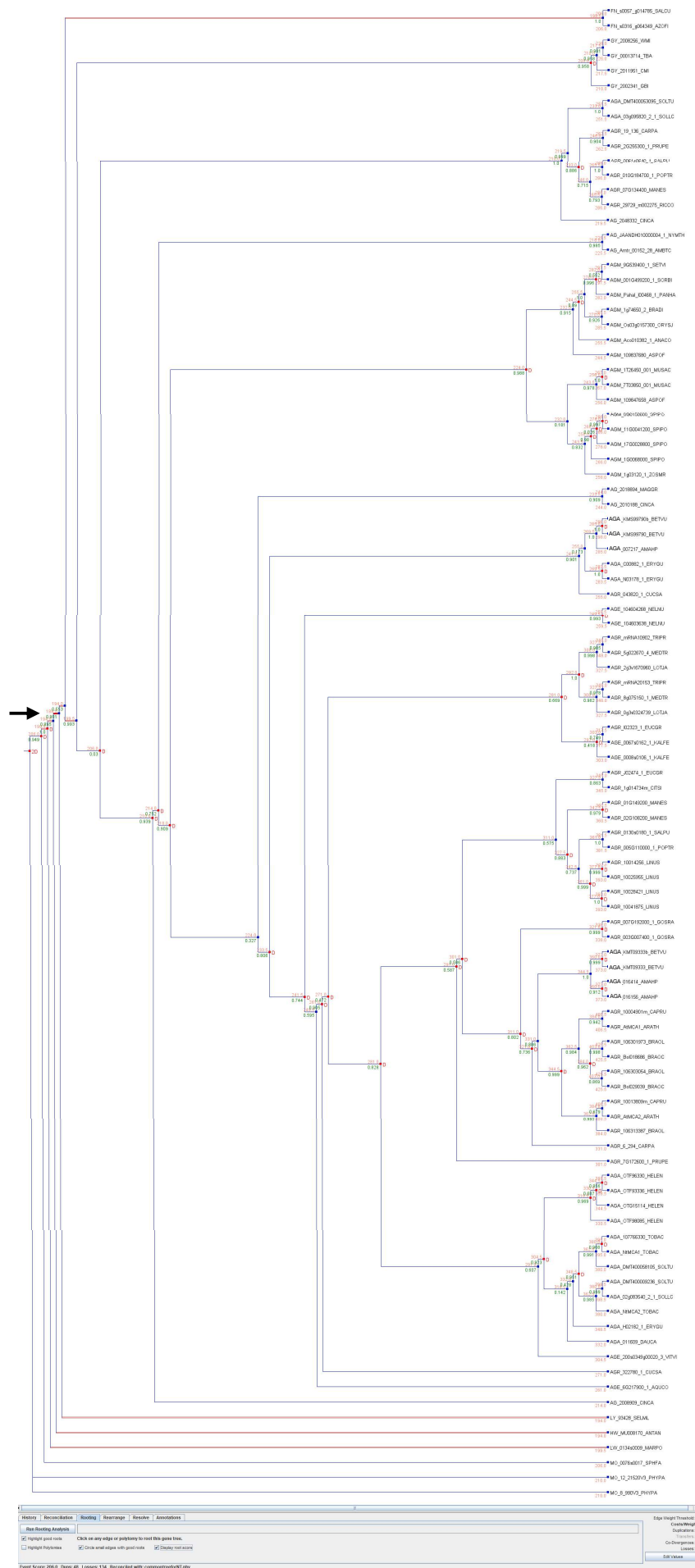

Supplement: S14 Appendix — (a) Results of the Notung rooting analyses of the MCA tree. Possible root position is marked in red (arrow). (b) Results of Notung rearrangement of the MCA tree. Rearranged branches are marked in yellow. D: inferred duplication. (PDF) [file pone.0249735.s014.pdf]
