# Supplementary material for: Mix and match: Patchwork domain evolution of the land plant-specific Ca2+-permeable mechanosensitive channel MCA
Source: PLoS One. 2021 Apr 15;16(4):e0249735. doi: 10.1371/journal.pone.0249735 (PMC8049495; doi:10.1371/journal.pone.0249735)

S6 Appendix. MCA gene found in the genome of *M. polymorpha* subsp. *ruderalis*.

Top: *M. polymorpha* cDNA 0134s0009.1, middle: *M. polymorpha* genome, bottom: *M. polymorpha* subsp. *ruderalis* genome

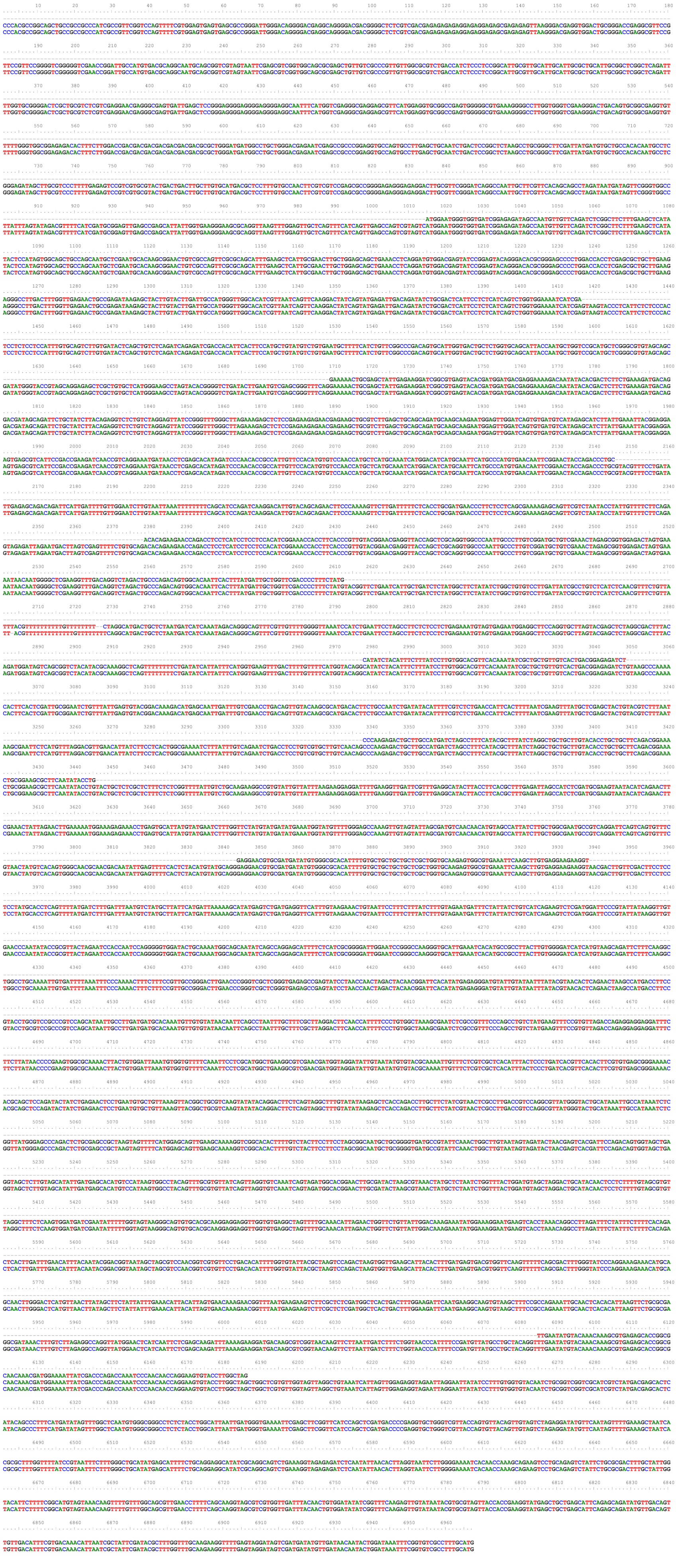

Supplement: S6 Appendix — Top: M. polymorpha cDNA 0134s0009.1, middle: M. polymorpha genome, bottom: M. polymorpha subsp. ruderalis genome. (PDF) [file pone.0249735.s006.pdf]
