# Supplementary material for: Mix and match: Patchwork domain evolution of the land plant-specific Ca2+-permeable mechanosensitive channel MCA
Source: PLoS One. 2021 Apr 15;16(4):e0249735. doi: 10.1371/journal.pone.0249735 (PMC8049495; doi:10.1371/journal.pone.0249735)
Supplement: S7 Appendix — (PDF) [file pone.0249735.s007.pdf]

S7 Appendix. MCA^{func} domain genes retrieved from proteomes.

taxon_no	taxon_id	protein_ID	length	E-value	E_score	target_description	gene_name
3	KLENI	A0A1Y1HMH1_KLENI	986	1.90E-17	63.2	U-box domain-containing protein	KFL_000380360
3	KLENI	A0A1Y1HNZ6_KLENI	1311	3.40E-15	55.9	U-box domain-containing protein	KFL_000210150
4	CHABU	A0A388K3W7_CHABU	929	1.50E-20	74.4	U-box domain-containing protein	CBR_g46708
4	CHABU	A0A388LWS0_CHABU	309	3.20E-17	63.6	Uncharacterized protein	CBR_g41724
4	CHABU	A0A388LEX4_CHABU	759	1.00E-11	45.7	U-box domain-containing protein	CBR_g31425
4	CHABU	A0A388LEZ4_CHABU	786	3.20E-10	40.8	U-box domain-containing protein	CBR_g31417
4	CHABU	A0A388LEW5_CHABU	706	3.10E-08	34.3	U-box domain-containing protein	CBR_g31414
4	CHABU	A0A388K3Y1_CHABU	1418	1.70E-07	31.9	Integrase catalytic domain-containing protein	CBR_g46709
4	CHABU	A0A388M6B9_CHABU	895	2.70E-06	28	U-box domain-containing protein	CBR_g50122
4	CHABU	A0A388KLL7_CHABU	421	8.60E-05	23.1	U-box domain-containing protein	CBR_g8241
5	MARPO	A0A2R6W7G4_MARPO	482	1.50E-61	206.9	Uncharacterized protein	MARPO_0134s0009
5	MARPO	A0A2R6XBS8_MARPO	447	8.30E-16	58.1	Phorbol-ester/DAG-type domain-containing protein	MARPO_0024s0070
5	MARPO	A0A2R6WB67_MARPO	853	1.10E-15	57.7	U-box domain-containing protein	MARPO_0115s0019
5	MARPO	A0A2R6XMC3_MARPO	1093	1.20E-09	38.1	U-box domain-containing protein	MARPO_0008s0036
5	MARPO	A0A2R6XUG2_MARPO	1001	3.70E-09	36.4	U-box domain-containing protein	MARPO_0002s0200
5	MARPO	A0A2R6X4Y8_MARPO	1014	6.10E-08	32.5	U-box domain-containing protein	MARPO_0036s0132
5	MARPO	A0A2R6WGW5_MARPO	491	1.40E-07	31.3	U-box domain-containing protein	MARPO_0092s0051
5	MARPO	A0A2R6XRA3_MARPO	524	3.00E-07	30.2	Uncharacterized protein	MARPO_0005s0262
5	MARPO	A0A2R6WAT9_MARPO	891	6.80E-06	25.8	U-box domain-containing protein	MARPO_0117s0025
6	MapoRu	A0A176VHI1_MARPO*	482	1.50E-61	206.9	Uncharacterized protein	AXG93_4905s1080: LVLJ01003617.1:83933-90898
6	MapoRu	A0A176VK07_MARPO	447	7.80E-16	58.1	Phorbol-ester/DAG-type domain-containing protein	AXG93_868s1360
6	MapoRu	A0A176WGY8_MARPO	853	1.00E-15	57.7	U-box domain-containing protein	AXG93_2294s1180
6	MapoRu	A0A176WDT8_MARPO	1093	1.10E-09	38.1	U-box domain-containing protein	AXG93_4876s1120
6	MapoRu	A0A176W0K6_MARPO	867	2.90E-09	36.7	Uncharacterized protein	AXG93_1712s1650
6	MapoRu	A0A176VN66_MARPO	1014	5.80E-08	32.5	U-box domain-containing protein	AXG93_2318s1000
6	MapoRu	A0A176VB93_MARPO	656	2.20E-07	30.6	U-box domain-containing protein	AXG93_3352s1000
6	MapoRu	A0A176WKN3_MARPO	524	2.90E-07	30.2	Uncharacterized protein	AXG93_4773s1550
6	MapoRu	A0A176VEC4_MARPO	842	3.60E-06	26.7	U-box domain-containing protein	AXG93_406s1240
7	PHYPA	A0A2K1JRK9_PHYPA	437	6.00E-71	238.2	Uncharacterized protein	PHYPA_016558
7	PHYPA	A0A2K1K5M7_PHYPA	444	1.50E-68	230.3	Uncharacterized protein	PHYPA_010980
7	PHYPA	A9SRL1_PHYPA	247	1.70E-67	226.9	Predicted protein	PHYPADRAFT_233853
7	PHYPA	A0A2K1KXP2_PHYPA	218	1.10E-51	175.5	Uncharacterized protein	PHYPA_005515
7	PHYPA	A0A2K1J288_PHYPA	681	5.90E-10	39.7	U-box domain-containing protein	PHYPA_021495
7	PHYPA	A0A2K1IUI4_PHYPA	654	1.20E-05	25.7	U-box domain-containing protein	PHYPA_024873
7	PHYPA	A0A2K1JTR6_PHYPA	1613	3.00E-05	24.4	Protein kinase domain-containing protein	PHYPA_014687
7	PHYPA	A0A2K1JM74_PHYPA	678	0.00021	21.7	U-box domain-containing protein	PHYPA_017471
7	PHYPA	A0A2K1KD90_PHYPA	1702	0.00023	21.5	Protein kinase domain-containing protein	PHYPA_010907
8	SELML	D8S521_SELML	380	5.00E-75	251.5	Uncharacterized protein	SELMODRAFT_418221
8	SELML	D8RGG8_SELML	395	1.80E-74	249.8	Uncharacterized protein	SELMODRAFT_93428
8	SELML	D8R2D4_SELML	1013	2.10E-11	44.6	U-box domain-containing protein	SELMODRAFT_167271
8	SELML	D8QT51_SELML	1014	2.40E-11	44.4	U-box domain-containing protein	SELMODRAFT_77566
8	SELML	D8RJG1_SELML	1002	1.10E-08	35.7	U-box domain-containing protein	SELMODRAFT_94734
8	SELML	D8QRE1_SELML	1002	1.20E-08	35.5	U-box domain-containing protein	SELMODRAFT_77081
8	SELML	D8SM19_SELML	986	1.50E-07	32	U-box domain-containing protein	SELMODRAFT_10232
8	SELML	D8RPB0_SELML	986	3.30E-07	30.9	U-box domain-containing protein	SELMODRAFT_10235
8	SELML	D8SS28_SELML	639	1.30E-06	29	U-box domain-containing protein	PUB13-1
8	SELML	D8SNN8_SELML	639	1.40E-06	28.9	U-box domain-containing protein	PUB13-2
8	SELML	D8RFG5_SELML	740	2.30E-06	28.2	U-box domain-containing protein	SELMODRAFT_410663
8	SELML	D8RVS0_SELML	684	8.60E-06	26.3	U-box domain-containing protein	PUB17-1
8	SELML	D8S2H5_SELML	684	9.30E-06	26.2	U-box domain-containing protein	PUB17-2
8	SELML	D8RK16_SELML	630	0.0005	20.6	U-box domain-containing protein	PUB12-1
8	SELML	D8SS39_SELML	1342	0.0005	20.5	Uncharacterized protein	SELMODRAFT_425129
8	SELML	D8SNP9_SELML	1342	0.00065	20.2	Uncharacterized protein	SELMODRAFT_446098
8	SELML	D8T3P6_SELML	768	0.00097	19.6	U-box domain-containing protein	PUB2-1
9	CMI	CMI00009383	425	5.00E-92	306.6	-	-
10	TBA	TBA00013714	420	1.80E-92	308.2	-	-
10	TBA	TBA00026688	76	1.50E-34	119.8	-	-
10	TBA	TBA00004295	826	6.60E-08	33.1	-	-
10	TBA	TBA00018241	668	1.30E-07	32.2	-	-
10	TBA	TBA00017111	608	3.60E-06	27.5	-	-
10	TBA	TBA00031826	628	2.70E-05	24.6	-	-
10	TBA	TBA00011918	158	0.00016	22.1	-	-
11	AMBTC	W1PMV8_AMBTC	421	3.30E-100	333.2	Uncharacterized protein	AMTR_s00152p00084120
11	AMBTC	W1NV09_AMBTC	1004	3.20E-09	37.2	RING-type E3 ubiquitin transferase	AMTR_s00002p00268880
11	AMBTC	W1PZ75_AMBTC	1013	4.70E-07	30.1	RING-type E3 ubiquitin transferase	AMTR_s00041p00118190
11	AMBTC	W1PD90_AMBTC	925	5.30E-06	26.7	Uncharacterized protein	AMTR_s00053p00044540
11	AMBTC	W1PIF9_AMBTC	803	6.40E-05	23.2	RING-type E3 ubiquitin transferase	AMTR_s00005p00254320
11	AMBTC	W1P688_AMBTC	700	0.00011	22.4	RING-type E3 ubiquitin transferase	AMTR_s00007p00217990
11	AMBTC	W1PMP7_AMBTC	665	0.00051	20.2	RING-type E3 ubiquitin transferase	AMTR_s00153p00035720
12	MUSAM	M0U802_MUSAM	423	6.10E-100	332.7	Uncharacterized protein	103973653
12	MUSAM	M0TEH5_MUSAM	415	4.50E-99	329.9	Uncharacterized protein	103990704
12	MUSAM	M0S3A2_MUSAM	418	2.00E-97	324.5	Uncharacterized protein	103998865
12	MUSAM	M0S725_MUSAM	545	5.80E-73	245	Protein kinase domain-containing protein	GSMUA_Achr2G113
12	MUSAM	M0TMJ7_MUSAM	983	9.10E-08	32.8	RING-type E3 ubiquitin transferase	GSMUA_Achr8G04440_001
12	MUSAM	M0S894_MUSAM	660	1.30E-05	25.8	RING-type E3 ubiquitin transferase	GSMUA_Achr2G15530_001
12	MUSAM	M0U4D9_MUSAM	599	4.60E-05	24.1	RING-type E3 ubiquitin transferase	GSMUA_Achr9G28820_001
12	MUSAM	M0TG32_MUSAM	638	9.40E-05	23.1	RING-type E3 ubiquitin transferase	GSMUA_Achr7G09440_001
12	MUSAM	M0SL69_MUSAM	950	0.00057	20.5	RING-type E3 ubiquitin transferase	GSMUA_Achr4G05020_001
13	ORYSJ	Q10RJ5_ORYSJ	418	9.60E-98	325.8	Mid1-complementing activity 1	OsMCA1
13	ORYSJ	A0A0P0Y563_ORYSJ	271	1.90E-44	152.5	Pkinase_Tyr domain-containing protein	Os11g0670100
13	ORYSJ	A0A0P0X0D3_ORYSJ	550	1.60E-41	142.9	Protein kinase domain-containing protein	Os06g0690200
13	ORYSJ	A0A0P0X0E5_ORYSJ	457	3.40E-38	132.1	Protein kinase domain-containing protein	Os06g0689600
13	ORYSJ	Q2QZT8_ORYSJ	214	7.50E-38	131	Expressed protein	Os11g0672300
13	ORYSJ	Q2R001_ORYSJ	543	5.20E-34	118.5	Protein kinase domain-containing protein	Os11g0664000
13	ORYSJ	A0A0N7KTB7_ORYSJ	219	1.10E-32	114.2	Os11g0666100 protein	Os11g0666100
13	ORYSJ	Q2QZY3_ORYSJ	537	5.70E-32	111.9	Protein kinase domain-containing protein	Os11g0666200
13	ORYSJ	Q2QZX6_ORYSJ	580	1.70E-31	110.3	Protein kinase domain-containing protein	Os11g0667000
13	ORYSJ	A0A0P0YAC0_ORYSJ	508	4.00E-31	109.1	Protein kinase domain-containing protein	Os12g0499700
13	ORYSJ	Q5VMJ7_ORYSJ	532	1.90E-30	106.9	Os06g0151700 protein	Os06g0151700
13	ORYSJ	Q5Z661_ORYSJ	540	6.10E-27	95.5	Os06g0693000 protein	Os06g0693000
13	ORYSJ	Q6ZDH7_ORYSJ	171	1.20E-26	94.6	Os08g0374701 protein	Os08g0374701
13	ORYSJ	A0A0P0Y569_ORYSJ	208	1.50E-25	91	Os11g0671950 protein	Os11g0671950
13	ORYSJ	A0A0N7KTB8_ORYSJ	423	1.70E-24	87.5	Protein kinase domain-containing protein	Os11g0667600
13	ORYSJ	A0A0N7KM87_ORYSJ	159	9.70E-24	85.1	Os06g0541600 protein	Os06g0541600
13	ORYSJ	Q0J6A8_ORYSJ	591	8.20E-21	75.5	Os08g0343000 protein	Os08g0343000
13	ORYSJ	B9G8M8_ORYSJ	354	1.10E-18	68.6	Os11g0668800 protein	Os11g0668800
13	ORYSJ	A0A0P0Y568_ORYSJ	167	2.70E-17	64.1	Os11g0664133 protein	Os11g0664133
13	ORYSJ	Q2QZU3_ORYSJ	279	5.90E-15	56.5	Expressed protein	LOC_Os11g44820
13	ORYSJ	Q2QZU0_ORYSJ	279	6.70E-13	49.8	Os11g0671700 protein	LOC_Os11g44850

taxon_no	taxon_id	protein_ID	length	E-value	E_score	target_description	gene_name
13	ORYSJ	A0A0P0Y5M9_ORYSJ	512	7.90E-13	49.6	Protein kinase domain-containing protein	Os11g0669200
13	ORYSJ	A0A0P0Y534_ORYSJ	77	1.30E-10	42.3	Os11g0665800 protein	Os11g0665800
13	ORYSJ	Q2QUY3_ORYSJ	654	9.10E-10	39.6	Protein kinase domain-containing protein	Os12g0249900
13	ORYSJ	A0A0P0Y580_ORYSJ	351	1.20E-09	39.2	Os11g0663800 protein	Os11g0663800
13	ORYSJ	Q2QV00_ORYSJ	130	5.00E-09	37.2	Os12g0248400 protein	LOC_Os12g14520
13	ORYSJ	Q2QZY8_ORYSJ	221	2.50E-07	31.7	Expressed protein	Os11g0665600
13	ORYSJ	Q6Z250_ORYSJ	642	5.50E-05	24.1	RING-type E3 ubiquitin transferase	Os08g0481200
13	ORYSJ	Q2QZZ6_ORYSJ	240	0.00041	21.2	Os11g0664400 protein	Os11g0664400
14	MAIZE	A0A1D6JP06_MAIZE	417	1.90E-97	324.7	Cell number regulator 13	Zm00001d027722
14	MAIZE	CNR13_MAIZE	428	2.00E-97	324.7	Cell number regulator 13	CNR13
14	MAIZE	A0A1D6HPN6_MAIZE	247	2.50E-92	308.1	Protein MID1-COMPLEMENTING ACTIVITY 1	Zm00001d018493
14	MAIZE	A0A1D6L6D3_MAIZE	411	1.90E-74	249.9	DNA mismatch repair protein MLH1	Zm00001d034247
14	MAIZE	A0A1D6PNG8_MAIZE	344	7.70E-41	140.6	Protein MID1-COMPLEMENTING ACTIVITY 1	100279975
14	MAIZE	A0A1D6LP67_MAIZE	410	3.30E-39	135.3	Protein kinase domain-containing protein	Zm00001d036532
14	MAIZE	A0A1D6F850_MAIZE	295	6.50E-34	118	Protein kinase domain-containing protein	Zm00001d007708
14	MAIZE	B4FE65_MAIZE	450	1.60E-15	58.2	Protein kinase domain-containing protein	100193199
14	MAIZE	A0A1D6KHR4_MAIZE	645	2.30E-06	28.4	RING-type E3 ubiquitin transferase	103641388
14	MAIZE	B6U243_MAIZE	461	5.50E-06	27.2	Protein kinase domain-containing protein	100277943
14	MAIZE	C0PG49_MAIZE	803	0.00093	19.9	RING-type E3 ubiquitin transferase	100383455
15	SORBI	C5WXT4_SORBI	420	1.60E-97	324.7	Uncharacterized protein	SORBI_3001G499200
15	SORBI	A0A1B6PJD7_SORBI	717	2.80E-45	154.8	Protein kinase domain-containing protein	SORBI_3006G008900
15	SORBI	A0A194YL77_SORBI	631	2.40E-40	138.8	Protein kinase domain-containing protein	SORBI_3010G242300
15	SORBI	C5Y7I4_SORBI	513	4.50E-40	137.9	Protein kinase domain-containing protein	SORBI_3005G209400
15	SORBI	A0A194YL48_SORBI	570	3.30E-39	135	Protein kinase domain-containing protein	SORBI_3010G242400
15	SORBI	A0A194YM61_SORBI	565	5.30E-38	131.1	Protein kinase domain-containing protein	SORBI_3010G239700
15	SORBI	C5Y7J4_SORBI	186	2.20E-35	122.6	Uncharacterized protein	SORBI_3005G209500
15	SORBI	A0A1Z5RKK0_SORBI	543	8.20E-28	98	Protein kinase domain-containing protein	SORBI_3005G210200
15	SORBI	A0A194YLS8_SORBI	525	1.30E-21	77.8	Protein kinase domain-containing protein	SORBI_3010G272300
15	SORBI	C5YQ08_SORBI	469	5.10E-20	72.6	Protein kinase domain-containing protein	SORBI_3008G002700
15	SORBI	A0A1B6PAJ6_SORBI	354	1.00E-07	32.6	Uncharacterized protein	SORBI_3009G252100
15	SORBI	C5YJR1_SORBI	643	1.10E-06	29.3	RING-type E3 ubiquitin transferase	SORBI_3007G226500
15	SORBI	A0A194YS10_SORBI	511	1.30E-06	29	Protein kinase domain-containing protein	SORBI_3004G212600
16	AQUCA	A0A2G5C987_AQUCA	429	1.70E-102	340.5	Uncharacterized protein	AQUCO_07500029v1
16	AQUCA	A0A2G5CUB3_AQUCA	1009	3.50E-08	33.6	RING-type E3 ubiquitin transferase	AQUCO_03700253v1
16	AQUCA	A0A2G5CME4_AQUCA	683	2.80E-06	27.5	RING-type E3 ubiquitin transferase	AQUCO_04500199v1
16	AQUCA	A0A2G5CJU1_AQUCA	1005	3.30E-06	27.2	RING-type E3 ubiquitin transferase	AQUCO_04900110v1
16	AQUCA	A0A2G5CST4_AQUCA	724	7.80E-06	26	RING-type E3 ubiquitin transferase	AQUCO_03900042v1
16	AQUCA	A0A2G5EM45_AQUCA	646	9.10E-06	25.8	RING-type E3 ubiquitin transferase	AQUCO_00700878v1
16	AQUCA	A0A2G5CR45_AQUCA	775	0.00052	20.1	RING-type E3 ubiquitin transferase	AQUCO_04000044v1
16	AQUCA	A0A2G5DCS2_AQUCA	1020	0.0028	17.7	RING-type E3 ubiquitin transferase	AQUCO_02200026v1
16	AQUCA	A0A2G5EAC6_AQUCA	762	0.0041	17.2	RING-type E3 ubiquitin transferase	AQUCO_01000513v1
16	AQUCA	A0A2G5DTG2_AQUCA	636	0.0099	15.9	RING-type E3 ubiquitin transferase	AQUCO_01500384v1
17	VITVI	F6H5I8_VITVI	420	3.20E-102	339.8	Uncharacterized protein	VIT_00s0349g00020
17	VITVI	F6HCB0_VITVI	688	6.70E-07	29.7	RING-type E3 ubiquitin transferase	VIT_13s0067g01460
17	VITVI	F6HMC1_VITVI	726	1.50E-06	28.6	RING-type E3 ubiquitin transferase	VIT_10s0003g01730
17	VITVI	F6HDW7_VITVI	1016	5.20E-06	26.9	RING-type E3 ubiquitin transferase	VIT_05s0020g04560
17	VITVI	E0CV89_VITVI	1003	1.80E-05	25.1	RING-type E3 ubiquitin transferase	VIT_16s0098g01320
17	VITVI	D7UCX6_VITVI	1006	0.00013	22.3	RING-type E3 ubiquitin transferase	VIT_15s0046g00020
17	VITVI	F6HJ78_VITVI	1019	0.0007	19.9	RING-type E3 ubiquitin transferase	VIT_01s0150g00330
18	POPTR	U5GEX3_POPTR	419	1.50E-101	338.2	Uncharacterized protein	POPTR_005G110000v3
18	POPTR	A0A2K1YWA1_POPTR	388	3.70E-58	197	Uncharacterized protein	POPTR_010G184700v3
18	POPTR	A0A3N7FL11_POPTR	1016	2.40E-08	34.9	RING-type E3 ubiquitin transferase	POPTR_010G079200
18	POPTR	A0A2K2BKD0_POPTR	1010	1.80E-07	32.1	RING-type E3 ubiquitin transferase	POPTR_002G175800v3
18	POPTR	A0A2K1Y7K4_POPTR	1004	5.00E-07	30.7	RING-type E3 ubiquitin transferase	POPTR_012G019900v3
18	POPTR	B9I1H0_POPTR	717	1.30E-06	29.4	RING-type E3 ubiquitin transferase	POPTR_011G071400v3
18	POPTR	B9HSL2_POPTR	688	5.70E-06	27.2	RING-type E3 ubiquitin transferase	POPTR_010G185900v3
18	POPTR	B9H2E3_POPTR	717	6.50E-05	23.8	RING-type E3 ubiquitin transferase	POPTR_004G061800v3
18	POPTR	A0A3N7FLZ4_POPTR	659	7.50E-05	23.6	RING-type E3 ubiquitin transferase	POPTR_009G165900
18	POPTR	B9I914_POPTR	1012	9.80E-05	23.2	RING-type E3 ubiquitin transferase	POPTR_014G102000v3
18	POPTR	B9GQ09_POPTR	658	0.0001	23.1	RING-type E3 ubiquitin transferase	POPTR_002G009900v3
18	POPTR	A0A2K1ZJ12_POPTR	775	0.00043	21.1	RING-type E3 ubiquitin transferase	POPTR_008G177100v3
19	MEDTR	G7LB65_MEDTR	424	9.30E-101	335.8	MID1-complementing activity-like protein	11445352
19	MEDTR	G7JXK8_MEDTR	416	2.20E-100	334.6	MID1-complementing activity-like protein	11421841
19	MEDTR	G7L516_MEDTR	1001	1.70E-07	32.4	RING-type E3 ubiquitin transferase	11435988
19	MEDTR	A0A072VMF8_MEDTR	1013	8.60E-07	30.1	RING-type E3 ubiquitin transferase	25484466
19	MEDTR	G7LAU3_MEDTR	1006	9.60E-06	26.7	RING-type E3 ubiquitin transferase	11440895
19	MEDTR	G7ILU6_MEDTR	720	7.00E-05	23.9	RING-type E3 ubiquitin transferase	11440815
19	MEDTR	G7JC16_MEDTR	766	0.00033	21.7	RING-type E3 ubiquitin transferase	11442685
19	MEDTR	PUB1_MEDTR	694	0.00046	21.3	U-box domain-containing protein 1	PUB1
19	MEDTR	G7KEX0_MEDTR	782	0.00078	20.5	RING-type E3 ubiquitin transferase	11412831
19	MEDTR	A0A072VQ10_MEDTR	630	0.0074	17.3	RING-type E3 ubiquitin transferase	25484932
20	CUCSA	A0A0A0L3N0_CUCSA	418	1.70E-101	337.2	Uncharacterized protein	Csa_3G002640
20	CUCSA	A0A0A0KGR0_CUCSA	336	1.20E-97	324.6	Uncharacterized protein	Csa_6G507150
20	CUCSA	A0A0A0K7R8_CUCSA	688	5.00E-09	36.4	RING-type E3 ubiquitin transferase	Csa_6G012250
20	CUCSA	A0A0A0KWP7_CUCSA	738	1.50E-06	28.3	RING-type E3 ubiquitin transferase	Csa_4G046720
20	CUCSA	A0A0A0LX59_CUCSA	715	4.60E-06	26.7	RING-type E3 ubiquitin transferase	Csa_1G467720
21	GOSRA	A0A0D2THW9_GOSRA	416	3.40E-102	340.1	Uncharacterized protein	B456_007G192000
21	GOSRA	A0A0D2MHL8_GOSRA	418	9.30E-102	338.7	Uncharacterized protein	B456_003G007400
21	GOSRA	A0A0D2S1Q5_GOSRA	1015	4.40E-08	33.9	RING-type E3 ubiquitin transferase	B456_006G245100
21	GOSRA	A0A0D2RZJ7_GOSRA	1011	4.20E-07	30.7	RING-type E3 ubiquitin transferase	B456_006G202700
21	GOSRA	A0A0D2SUC9_GOSRA	711	5.40E-07	30.4	RING-type E3 ubiquitin transferase	B456_006G089900
21	GOSRA	A0A0D2QQY2_GOSRA	1006	1.80E-06	28.7	RING-type E3 ubiquitin transferase	B456_001G152800
21	GOSRA	A0A0D2VB40_GOSRA	720	3.60E-06	27.7	RING-type E3 ubiquitin transferase	B456_010G161700
21	GOSRA	A0A0D2Q4J9_GOSRA	645	9.60E-06	26.3	RING-type E3 ubiquitin transferase	B456_002G116600
21	GOSRA	A0A0D2VT35_GOSRA	623	2.00E-05	25.3	RING-type E3 ubiquitin transferase	B456_011G291400
21	GOSRA	A0A0D2UNH3_GOSRA	711	3.30E-05	24.6	RING-type E3 ubiquitin transferase	B456_011G036500
21	GOSRA	A0A0D2QNN8_GOSRA	669	0.0001	23	RING-type E3 ubiquitin transferase	B456_001G109500
21	GOSRA	A0A0D2NIA8_GOSRA	640	0.0003	21.5	RING-type E3 ubiquitin transferase	B456_005G258700
21	GOSRA	A0A0D2R9W1_GOSRA	630	0.00046	20.8	RING-type E3 ubiquitin transferase	B456_002G206200
21	GOSRA	A0A0D2UXB8_GOSRA	637	0.0007	20.3	RING-type E3 ubiquitin transferase	B456_011G237300
22	BRAOL	A0A0D3A1N3_BRAOL	420	1.10E-101	339.1	Uncharacterized protein	106303054
22	BRAOL	A0A0D3DHX0_BRAOL	412	5.80E-100	333.5	Uncharacterized protein	106301973
22	BRAOL	A0A0D3E374_BRAOL	411	3.20E-98	327.8	Uncharacterized protein	Bo9g024720
22	BRAOL	A0A0D3BIU0_BRAOL	718	0.00019	22.7	RING-type E3 ubiquitin transferase	106335240
22	BRAOL	A0A0D3D625_BRAOL	711	0.00042	21.6	RING-type E3 ubiquitin transferase	106306796
22	BRAOL	A0A0D3A8T5_BRAOL	661	0.00058	21.1	RING-type E3 ubiquitin transferase	106310349
22	BRAOL	A0A0D3DHX6_BRAOL	658	0.00074	20.8	Uncharacterized protein	106306374
23	ARATH	MCAC1_ARATH	421	5.90E-101	335.6	Protein MID1-COMPLEMENTING ACTIVITY 1	MCA1
23	ARATH	MCAC2_ARATH	416	9.50E-92	305.7	Protein MID1-COMPLEMENTING ACTIVITY 2	MCA2
23	ARATH	PUB13_ARATH	660	0.00019	21.7	U-box domain-containing protein 13	PUB13
23	ARATH	O65640_ARATH	670	0.0003	21	Armadillo repeat only 3	ARO3
23	ARATH	PUB45_ARATH	768	0.00031	21	U-box domain-containing protein 45	PUB45

taxon_no	taxon_id	protein_ID	length	E-value	E_score	target_description	gene_name
24	ERYGU	A0A022QT97_ERYGU	413	1.00E-96	321.8	Uncharacterized protein	MIMGU_mgv1a007258mg
24	ERYGU	A0A022RAP2_ERYGU	295	1.90E-92	308	Uncharacterized protein	MIMGU_mgv1a011010mg
24	ERYGU	A0A022PTL3_ERYGU	404	8.10E-91	302.7	Uncharacterized protein	MIMGU_mgv1a007522mg
24	ERYGU	A0A022PX08_ERYGU	663	2.90E-11	43.8	RING-type E3 ubiquitin transferase	MIMGU_mgv1a025209mg
24	ERYGU	A0A022Q8X8_ERYGU	1024	8.70E-11	42.3	U-box domain-containing protein	MIMGU_mgv1a026838mg
24	ERYGU	A0A022R0E6_ERYGU	1013	1.20E-06	28.8	RING-type E3 ubiquitin transferase	MIMGU_mgv1a025157mg
24	ERYGU	A0A022QHE9_ERYGU	700	5.70E-06	26.6	RING-type E3 ubiquitin transferase	MIMGU_mgv1a027038mg
24	ERYGU	A0A022RCR4_ERYGU	648	5.70E-05	23.4	RING-type E3 ubiquitin transferase	MIMGU_mgv1a002670mg
24	ERYGU	A0A022RWG3_ERYGU	711	0.00031	21	RING-type E3 ubiquitin transferase	MIMGU_mgv1a002121mg
25	SOLLC	A0A3Q7F6L0_SOLLC	418	1.10E-98	328.6	Uncharacterized protein	101266587
25	SOLLC	A0A3Q7FPG3_SOLLC	738	8.90E-53	179.2	PMR5N domain-containing protein	Solyc03g095820.3
25	SOLLC	A0A3Q7I6W5_SOLLC	1015	2.00E-08	34.9	RING-type E3 ubiquitin transferase	Solyc09g083060.3
25	SOLLC	A0A3Q7FMR7_SOLLC	1007	1.30E-05	25.8	RING-type E3 ubiquitin transferase	100316887
25	SOLLC	A0A3Q7F380_SOLLC	724	3.50E-05	24.4	RING-type E3 ubiquitin transferase	101245319
25	SOLLC	A0A3Q7GT14_SOLLC	637	3.70E-05	24.3	RING-type E3 ubiquitin transferase	Solyc06g051090.2
25	SOLLC	A0A3Q7G8J9_SOLLC	649	0.00036	21.1	RING-type E3 ubiquitin transferase	101259042
