# Supplementary material for: Mix and match: Patchwork domain evolution of the land plant-specific Ca2+-permeable mechanosensitive channel MCA
Source: PLoS One. 2021 Apr 15;16(4):e0249735. doi: 10.1371/journal.pone.0249735 (PMC8049495; doi:10.1371/journal.pone.0249735)
Supplement: S8 Appendix — (PDF) [file pone.0249735.s008.pdf]

S8 Appendix. PLAC8 domain genes retrieved from proteomes.

taxon_no	taxon_id	target_name	length	E-value	E_score	target_description	gene_name
1	CHLRE	Q9M5D4_CHLRE	241	2.50E-21	76.5	Early zygote protein	Class VIII
1	CHLRE	A8J0X1_CHLRE	254	2.50E-20	73.3	Predicted protein	CHLRE_02g145900v5
1	CHLRE	A0A2K3E251_CHLRE	434	2.50E-20	73.3	Uncharacterized protein	CHLRE_02g098500v5
1	CHLRE	A0A2K3CRI8_CHLRE	201	9.80E-19	68.2	Uncharacterized protein	CHLRE_17g738000v5
1	CHLRE	A0A2K3E223_CHLRE	284	1.20E-18	67.9	Uncharacterized protein	CHLRE_02g097150v5
1	CHLRE	A0A2K3E033_CHLRE	146	5.50E-18	65.8	Uncharacterized protein	CHLRE_02g075000v5
1	CHLRE	A0A2K3CRJ7_CHLRE	137	2.70E-17	63.6	Uncharacterized protein	CHLRE_17g738050v5
1	CHLRE	A0A2K3DVZ9_CHLRE	345	1.20E-16	61.5	Uncharacterized protein	CHLRE_03g155527v5
1	CHLRE	A0A2K3DFG0_CHLRE	126	1.90E-16	60.8	Uncharacterized protein	CHLRE_09g408800v5
1	CHLRE	A8JBM3_CHLRE	176	2.20E-14	54.2	Predicted protein	CHLRE_16g695700v5
1	CHLRE	A0A2K3E051_CHLRE	220	2.30E-14	54.2	Uncharacterized protein	CHLRE_02g075400v5
1	CHLRE	A0A2K3D2G8_CHLRE	1160	7.30E-08	33.3	Uncharacterized protein	CHLRE_12g512450v5
2	VOLCA	D8TN15_VOLCA	114	3.30E-20	72.6	Uncharacterized protein	VOLCADRAFT_103596
2	VOLCA	D8TWA4_VOLCA	343	4.90E-20	72	Uncharacterized protein	VOLCADRAFT_91142
2	VOLCA	D8UIG1_VOLCA	1221	3.30E-17	63	Pkinase_Tyr domain-containing protein	VOLCADRAFT_108130
2	VOLCA	D8TZP3_VOLCA	205	4.40E-17	62.6	Uncharacterized protein	VOLCADRAFT_105270
2	VOLCA	D8TN09_VOLCA	231	1.30E-14	54.7	Uncharacterized protein	VOLCADRAFT_103592
2	VOLCA	D8U0U6_VOLCA	168	5.20E-14	52.7	Uncharacterized protein	VOLCADRAFT_105442
2	VOLCA	D8TPB0_VOLCA	1022	3.40E-05	24.5	Uncharacterized protein	VOLCADRAFT_88551
3	KLENI	A0A1Y1HJ22_KLENI	195	7.70E-26	90.8	Uncharacterized protein	KFL_000050400
3	KLENI	A0A1Y1HNA6_KLENI	187	1.30E-25	90.1	Uncharacterized protein	KFL_000460310
3	KLENI	A0A0U9HJG6_KLENI	183	1.20E-24	87.1	Uncharacterized protein	KFL_000920270
3	KLENI	A0A1Y1I832_KLENI	288	4.90E-24	85.1	PLAC8 family protein	KFL_002080240
3	KLENI	A0A1Y1I3S2_KLENI	178	1.40E-23	83.6	Uncharacterized protein	KFL_002420170
3	KLENI	A0A1Y1I596_KLENI	407	6.00E-22	78.4	Uncharacterized protein	KFL_001960060
3	KLENI	A0A1Y1I5Z5_KLENI	241	6.50E-21	75.1	PLAC8 family protein	KFL_002080060
3	KLENI	A0A1Y1IAP5_KLENI	239	3.50E-17	63.1	Uncharacterized protein	KFL_003950120
3	KLENI	A0A1Y1IIJ9_KLENI	211	1.20E-16	61.4	Uncharacterized protein	KFL_006640040
3	KLENI	A0A1Y1HMQ7_KLENI	575	2.70E-14	53.8	PLAC8 family protein	KFL_000100460
3	KLENI	A0A1Y1I3Z6_KLENI	197	8.10E-12	45.9	Uncharacterized protein	KFL_001480250
3	KLENI	A0A1Y1HSG6_KLENI	141	9.10E-06	26.5	Uncharacterized protein	KFL_000460300
3	KLENI	A0A1Y1IDC3_KLENI	373	0.00027	21.7	Uncharacterized protein	KFL_004740050
4	CHABU	A0A388L496_CHABU	212	9.10E-25	88.5	Uncharacterized protein	CBR_g23456
4	CHABU	A0A388JTR3_CHABU	326	4.90E-24	86.1	Uncharacterized protein	CBR_g19739
4	CHABU	A0A388LZY7_CHABU	231	7.40E-23	82.3	Uncharacterized protein	CBR_g45935
4	CHABU	A0A388L492_CHABU	228	1.00E-22	81.9	Uncharacterized protein	CBR_g23455
4	CHABU	A0A388K699_CHABU	163	5.00E-21	76.5	Uncharacterized protein	CBR_g51429
4	CHABU	A0A388L8L5_CHABU	126	4.30E-20	73.5	Uncharacterized protein	CBR_g27758
4	CHABU	A0A388K0I1_CHABU	358	8.30E-18	66.1	Uncharacterized protein	CBR_g38081
4	CHABU	A0A388K675_CHABU	290	3.30E-17	64.2	Uncharacterized protein	CBR_g51437
4	CHABU	A0A388M6T3_CHABU	355	1.30E-16	62.3	Uncharacterized protein	CBR_g50374
4	CHABU	A0A388JYZ7_CHABU	354	2.10E-14	55.2	Uncharacterized protein	CBR_g34304
4	CHABU	A0A388K6A4_CHABU	131	4.60E-13	50.9	Uncharacterized protein	CBR_g51444
4	CHABU	A0A388K686_CHABU	778	2.10E-09	39.2	Uncharacterized protein	CBR_g51446
5	MARPO	A0A2R6XBX3_MARPO	195	1.60E-25	90	Uncharacterized protein	MARPO_0024s0111
5	MARPO	A0A2R6XHS0_MARPO	152	2.70E-25	89.3	Uncharacterized protein	MARPO_0014s0163
5	MARPO	A0A2R6XME1_MARPO	159	3.50E-23	82.5	Uncharacterized protein	MARPO_0008s0050
5	MARPO	A0A2R6XDZ4_MARPO	327	7.10E-23	81.6	Uncharacterized protein	MARPO_0020s0004
5	MARPO	A0A2R6WWS2_MARPO	232	1.30E-21	77.5	Uncharacterized protein	MARPO_0052s0073
5	MARPO	A0A2R6WMU3_MARPO	151	1.50E-21	77.3	Uncharacterized protein	MARPO_0073s0040
5	MARPO	A0A2R6XDZ2_MARPO	150	2.10E-21	76.9	Uncharacterized protein	MARPO_0020s0003
5	MARPO	A0A2R6XSH1_MARPO	159	7.20E-21	75.1	Uncharacterized protein	MARPO_0004s0278
5	MARPO	A0A2R6W0U0_MARPO	157	9.90E-19	68.3	Uncharacterized protein	MARPO_0197s0005
5	MARPO	A0A2R6WJJ2_MARPO	879	9.60E-18	65.1	Uncharacterized protein	MARPO_0084s0076
5	MARPO	A0A2R6W7G4_MARPO	482	5.50E-15	56.3	Uncharacterized protein	MARPO_0134s0009
5	MARPO	A0A2R6W3Y7_MARPO	161	1.90E-08	35.3	Uncharacterized protein	MARPO_0161s0015
5	MARPO	A0A2R6W2H6_MARPO	128	5.80E-07	30.5	Uncharacterized protein	MARPO_0175s0012
6	MapoRu	A0A176VHI1_MARPO*	482	5.50E-15	56.3	Uncharacterized protein	AXG93_4905s1080: LVLJ01003617.1:83933-90898
6	MapoRu	A0A176VYM7_MARPO	195	1.50E-25	90	Uncharacterized protein	AXG93_4620s1090
6	MapoRu	A0A176WCA6_MARPO	232	1.20E-21	77.5	Uncharacterized protein	AXG93_3893s1210
6	MapoRu	A0A176W3Z0_MARPO	159	6.70E-21	75.1	Uncharacterized protein	AXG93_4666s1360
6	MapoRu	A0A176VZ02_MARPO	388	2.50E-20	73.3	Uncharacterized protein	AXG93_4601s1400
6	MapoRu	A0A176WKB6_MARPO	838	3.00E-18	66.6	Uncharacterized protein	AXG93_374s1090
6	MapoRu	A0A176VKN2_MARPO	161	1.80E-08	35.3	Uncharacterized protein	AXG93_2338s1150
7	PHYPA	A0A2K1JCB4_PHYPA	224	4.80E-26	92.4	Uncharacterized protein	PHYPA_019450
7	PHYPA	A0A2K1J512_PHYPA	180	9.60E-26	91.4	Uncharacterized protein	PHYPA_022468
7	PHYPA	A0A2K1K2N2_PHYPA	225	1.00E-25	91.4	Uncharacterized protein	PHYPA_012510
7	PHYPA	A9TU75_PHYPA	196	2.30E-25	90.2	Predicted protein	PHYPADRAFT_198329
7	PHYPA	A0A2K1KSR6_PHYPA	210	2.50E-25	90.1	Uncharacterized protein	PHYPA_003817
7	PHYPA	A0A2K1LBB4_PHYPA	184	5.80E-24	85.7	Uncharacterized protein	PHYPA_001741
7	PHYPA	A9RSV7_PHYPA	234	9.10E-24	85.1	Predicted protein	PHYPADRAFT_205311
7	PHYPA	A9T9E6_PHYPA	142	1.00E-23	85	Predicted protein	PHYPADRAFT_192851
7	PHYPA	A9RYB4_PHYPA	141	2.50E-22	80.5	Predicted protein	PHYPADRAFT_179434
7	PHYPA	A9TS84_PHYPA	223	2.10E-21	77.5	Predicted protein	PHYPADRAFT_197685
7	PHYPA	A0A2K1JS16_PHYPA	326	1.30E-19	71.8	Uncharacterized protein	PHYPA_016704
7	PHYPA	A0A2K1IQL3_PHYPA	220	1.30E-19	71.7	Uncharacterized protein	PHYPA_025692
7	PHYPA	A9RQI4_PHYPA	231	2.00E-17	64.8	Predicted protein	PHYPADRAFT_160465
7	PHYPA	A0A2K1IQE0_PHYPA	289	2.20E-17	64.6	Uncharacterized protein	PHYPA_025616
7	PHYPA	A0A2K1J0A7_PHYPA	729	3.20E-17	64.1	Uncharacterized protein	PHYPA_022865
7	PHYPA	A0A2K1J1P2_PHYPA	259	4.10E-17	63.8	Uncharacterized protein	PHYPA_023348
7	PHYPA	A0A2K1IRB4_PHYPA	696	4.30E-16	60.5	Uncharacterized protein	PHYPA_025943
7	PHYPA	A0A2K1KPG7_PHYPA	594	2.10E-15	58.3	Uncharacterized protein	PHYPA_006538
7	PHYPA	A0A2K1JFW5_PHYPA	565	4.40E-14	54	Uncharacterized protein	PHYPA_017825
7	PHYPA	A0A2K1JRK9_PHYPA	437	1.00E-13	52.9	Uncharacterized protein	PHYPA_016558
7	PHYPA	A0A2K1K5M7_PHYPA	444	1.40E-12	49.2	Uncharacterized protein	PHYPA_010980
7	PHYPA	A0A2K1JMQ8_PHYPA	150	5.80E-10	40.8	Uncharacterized protein	PHYPA_017661
7	PHYPA	A0A2K1JM10_PHYPA	167	3.20E-09	38.4	Uncharacterized protein	PHYPA_017414
8	SELML	D8QTV8_SELML	123	1.20E-26	94.5	Uncharacterized protein	SELMODRAFT_68870
8	SELML	D8SVI0_SELML	227	3.30E-25	89.8	Uncharacterized protein	SELMODRAFT_125873
8	SELML	D8T0N0_SELML	227	3.30E-25	89.8	Uncharacterized protein	SELMODRAFT_159960
8	SELML	D8RJX1_SELML	118	2.00E-24	87.3	Uncharacterized protein	SELMODRAFT_38123
8	SELML	D8R4S8_SELML	116	2.50E-24	87	Uncharacterized protein	SELMODRAFT_69283
8	SELML	D8R4T1_SELML	116	2.50E-24	87	Uncharacterized protein	SELMODRAFT_69284
8	SELML	D8SQZ8_SELML	117	3.30E-24	86.6	Uncharacterized protein	SELMODRAFT_122730
8	SELML	D8R4S6_SELML	171	3.70E-23	83.2	Uncharacterized protein	SELMODRAFT_439474
8	SELML	D8QYQ3_SELML	105	1.10E-20	75.3	Uncharacterized protein	SELMODRAFT_28363
8	SELML	D8RGG8_SELML	395	4.50E-17	63.7	Uncharacterized protein	SELMODRAFT_93428
8	SELML	D8SWF2_SELML	363	5.50E-16	60.2	Uncharacterized protein	SELMODRAFT_43443
8	SELML	D8SIL9_SELML	374	5.90E-16	60.1	Uncharacterized protein	SELMODRAFT_43451
8	SELML	D8S521_SELML	380	4.50E-15	57.3	Uncharacterized protein	SELMODRAFT_418221
8	SELML	D8R4S4_SELML	808	1.30E-11	46.3	Uncharacterized protein	SELMODRAFT_407356
8	SELML	D8R8C2_SELML	64	1.30E-05	27	Uncharacterized protein	SELMODRAFT_87327
9	CMI	CMI00028146	237	1.20E-24	87.8	-	-
9	CMI	CMI00025731	115	1.70E-21	77.8	-	-
9	CMI	CMI00009868	165	6.00E-21	76	-	-
9	CMI	CMI00028134	162	1.90E-20	74.3	-	-
9	CMI	CMI00009383	425	8.80E-15	56.2	-	-
9	CMI	CMI00023193	119	5.30E-13	50.5	-	-
9	CMI	CMI00025911	163	9.10E-13	49.7	-	-
9	CMI	CMI00013570	108	1.10E-12	49.5	-	-
9	CMI	CMI00000599	76	9.90E-09	36.8	-	-

taxon_no	taxon_id	target_name	length	E-value	E_score	target_description	gene_name
9	CMI	CMI00012407	92	7.20E-08	34	-	-
9	CMI	CMI00015584	50	4.00E-06	28.4	-	-
9	CMI	CMI00012067	157	7.80E-06	27.5	-	-
10	TBA	TBA00030604	238	1.70E-24	87.5	-	-
10	TBA	TBA00030323	282	2.80E-24	86.8	-	-
10	TBA	TBA00029963	192	7.90E-24	85.3	-	-
10	TBA	TBA00008094	218	3.20E-23	83.4	-	-
10	TBA	TBA00019197	176	9.00E-22	78.8	-	-
10	TBA	TBA00015982	135	1.20E-21	78.4	-	-
10	TBA	TBA00008099	227	2.80E-19	70.7	-	-
10	TBA	TBA00021789	675	2.60E-18	67.6	-	-
10	TBA	TBA00013714	420	7.90E-14	53.3	-	-
10	TBA	TBA00012778	68	2.10E-09	39.1	-	-
11	AMBTC	W1NEZ9_AMBTC	191	2.00E-27	96.7	Uncharacterized protein	AMTR_s00010p00034340
11	AMBTC	W1NZK6_AMBTC	238	5.20E-25	88.9	Uncharacterized protein	AMTR_s00106p00154260
11	AMBTC	W1NFT2_AMBTC	162	1.40E-24	87.6	Uncharacterized protein	AMTR_s00010p00033430
11	AMBTC	U5CZS5_AMBTC	139	8.90E-24	85	Uncharacterized protein	AMTR_s00067p00142050
11	AMBTC	W1NFR7_AMBTC	258	1.80E-20	74.4	Uncharacterized protein	AMTR_s00010p00032660
11	AMBTC	W1NTE2_AMBTC	207	1.70E-18	68	Uncharacterized protein	AMTR_s00072p00053710
11	AMBTC	W1NKP1_AMBTC	236	1.30E-17	65.2	Uncharacterized protein	AMTR_s00023p00246550
11	AMBTC	W1PCC6_AMBTC	567	6.80E-17	62.9	Uncharacterized protein	AMTR_s00007p00197420
11	AMBTC	W1NU01_AMBTC	189	9.30E-17	62.4	Uncharacterized protein	AMTR_s00101p00073580
11	AMBTC	W1PMV8_AMBTC	421	4.90E-14	53.7	Uncharacterized protein	AMTR_s00152p00084120
12	MUSAM	M0U3Y7_MUSAM	175	1.00E-28	101.2	Uncharacterized protein	103999184
12	MUSAM	M0TIR3_MUSAM	168	1.60E-28	100.5	Uncharacterized protein	GSMUA_Achr7G18760_001
12	MUSAM	M0UA62_MUSAM	184	2.80E-26	93.4	Uncharacterized protein	103974361
12	MUSAM	M0TIR0_MUSAM	182	3.50E-26	93.1	Uncharacterized protein	103992193
12	MUSAM	M0TIR2_MUSAM	182	1.00E-25	91.5	Uncharacterized protein	103992194
12	MUSAM	M0T3H6_MUSAM	182	1.30E-25	91.2	Uncharacterized protein	103986725
12	MUSAM	M0TC06_MUSAM	188	1.20E-24	88.1	Uncharacterized protein	103989638
12	MUSAM	M0RIC1_MUSAM	418	5.90E-24	85.9	Uncharacterized protein	GSMUA_Achr10G14950_001
12	MUSAM	M0T0X1_MUSAM	269	7.90E-24	85.5	Uncharacterized protein	GSMUA_Achr5G22960_001
12	MUSAM	M0RPD9_MUSAM	233	1.90E-23	84.3	Uncharacterized protein	GSMUA_Achr11G04590_001
12	MUSAM	M0RWZ2_MUSAM	709	2.60E-23	83.9	Uncharacterized protein	GSMUA_Achr1G04340_001
12	MUSAM	M0TFD8_MUSAM	238	4.00E-23	83.3	Uncharacterized protein	103990416
12	MUSAM	M0RHU2_MUSAM	244	1.50E-21	78.2	Uncharacterized protein	104000945
12	MUSAM	M0T7A2_MUSAM	256	1.10E-19	72.2	Uncharacterized protein	103987911
12	MUSAM	M0U8Z7_MUSAM	244	1.10E-18	69	Uncharacterized protein	GSMUA_AchrUn_randomG13880_001
12	MUSAM	M0RTQ2_MUSAM	551	2.20E-17	64.8	Uncharacterized protein	GSMUA_Achr11G19720_001
12	MUSAM	M0U8G5_MUSAM	547	5.00E-17	63.7	Uncharacterized protein	GSMUA_AchrUn_randomG12060_001
12	MUSAM	M0S3A2_MUSAM	418	1.10E-16	62.6	Uncharacterized protein	103998865
12	MUSAM	M0S4P0_MUSAM	301	2.30E-16	61.6	Uncharacterized protein	GSMUA_Achr2G02990_001
12	MUSAM	M0TEH5_MUSAM	415	3.50E-16	61	Uncharacterized protein	103990704
12	MUSAM	M0TXM7_MUSAM	153	4.90E-15	57.3	Uncharacterized protein	GSMUA_Achr9G05180_001
12	MUSAM	M0U802_MUSAM	423	3.90E-14	54.4	Uncharacterized protein	103973653
12	MUSAM	M0RTQ3_MUSAM	505	1.60E-09	39.6	Uncharacterized protein	GSMUA_Achr11G19730_001
12	MUSAM	M0TJR1_MUSAM	176	1.70E-09	39.5	Uncharacterized protein	GSMUA_Achr7G22250_001
12	MUSAM	M0TJR3_MUSAM	176	4.30E-09	38.3	Uncharacterized protein	GSMUA_Achr7G22270_001
12	MUSAM	M0RR50_MUSAM	937	3.70E-07	32	Uncharacterized protein	GSMUA_Achr11G10700_001
13	ORYSJ	Q850Y6_ORYSJ	150	2.30E-29	103.5	Expressed protein	Os03g0830200
13	ORYSJ	A0A0P0VL00_ORYSJ	192	3.50E-28	99.8	Os02g0580000 protein	Os02g0580000
13	ORYSJ	Q6EP48_ORYSJ	162	3.60E-28	99.7	Os02g0579800 protein	Os02g0579800
13	ORYSJ	Q850Y8_ORYSJ	148	3.70E-28	99.7	Os03g0830400 protein	Os03g0830400
13	ORYSJ	Q0JCM6_ORYSJ	179	1.30E-27	97.9	Os04g0461600 protein	Os04g0461600
13	ORYSJ	A0A0N7KLW5_ORYSJ	233	8.80E-27	95.3	Os06g0266266 protein	Os06g0266266
13	ORYSJ	Q850Y9_ORYSJ	141	3.40E-26	93.4	Os03g0830500 protein	Os03g0830500
13	ORYSJ	Q6Z6F9_ORYSJ	181	1.50E-25	91.3	Os02g0763000 protein	Os02g0763000
13	ORYSJ	Q33BF6_ORYSJ	186	2.50E-24	87.4	Os10g0112100 protein	Os10g0112100
13	ORYSJ	Q850Y3_ORYSJ	136	5.80E-23	83	Os03g0829900 protein	Os03g0829900
13	ORYSJ	Q10T45_ORYSJ	257	2.60E-22	80.9	Os03g0101800 protein	Os03g0101800
13	ORYSJ	A0A0P0WXH6_ORYSJ	113	8.10E-22	79.3	Os06g0520733 protein	Os06g0520733
13	ORYSJ	Q0IVQ1_ORYSJ	235	8.60E-22	79.2	Os10g0560200 protein	Os10g0560200
13	ORYSJ	Q9FYQ0_ORYSJ	235	6.40E-21	76.4	ESTs AU075439(E60711)	Os01g0157900
13	ORYSJ	Q94D92_ORYSJ	525	2.30E-19	71.5	Os01g0825900 protein	Os01g0825900
13	ORYSJ	Q10MQ4_ORYSJ	610	3.30E-19	71	Os03g0299800 protein	Os03g0299800
13	ORYSJ	B7F9H9_ORYSJ	147	8.80E-19	69.6	Os01g0267400 protein	Os01g0267400
13	ORYSJ	Q65WW0_ORYSJ	554	7.20E-18	66.7	Os05g0474900 protein	Os05g0474900
13	ORYSJ	H2KW45_ORYSJ	491	1.20E-17	65.9	Os11g0109633 protein	Os11g0109633
13	ORYSJ	Q2RBJ0_ORYSJ	553	1.80E-16	62.2	Os11g0109700 protein	Os11g0109700
13	ORYSJ	Q5W6P5_ORYSJ	521	2.20E-16	61.9	Os05g0341900 protein	Os05g0341900
13	ORYSJ	Q10RJ5_ORYSJ	418	3.20E-16	61.4	Mid1-complementing activity 1	OsMCA1
13	ORYSJ	Q850Y7_ORYSJ	166	6.10E-13	50.9	Os03g0830300 protein	Os03g0830300
14	MAIZE	CNR2_MAIZE	181	6.20E-28	98.8	Cell number regulator 2	CNR2
14	MAIZE	A0A1D6H0Y8_MAIZE	138	7.50E-28	98.5	Protein PLANT CADMIUM RESISTANCE 11	Zm00001d015276
14	MAIZE	CNR10_MAIZE	157	2.10E-27	97.1	Cell number regulator 10	CNR10
14	MAIZE	CNR11_MAIZE	158	8.60E-27	95.1	Cell number regulator 11	CNR11
14	MAIZE	CNR7_MAIZE	180	2.80E-26	93.5	Cell number regulator 7	CNR7
14	MAIZE	A0A1X7YED2_MAIZE	144	6.80E-26	92.3	Uncharacterized protein	100500711
14	MAIZE	CNR4_MAIZE	159	8.20E-26	92	Cell number regulator 4	CNR4
14	MAIZE	CNR9_MAIZE	175	8.70E-26	91.9	Cell number regulator 9	CNR9
14	MAIZE	CNR5_MAIZE	184	2.50E-25	90.5	Cell number regulator 5	CNR5
14	MAIZE	CNR3_MAIZE	167	4.70E-25	89.6	Cell number regulator 3	CNR3
14	MAIZE	K7UKY3_MAIZE	189	3.50E-24	86.8	Cell number regulator 1	Zm00001d052171
14	MAIZE	CNR1_MAIZE	191	3.60E-24	86.7	Cell number regulator 1	CNR1
14	MAIZE	CNR6_MAIZE	239	3.10E-22	80.5	Cell number regulator 6	CNR6
14	MAIZE	CNR8_MAIZE	233	2.10E-21	77.9	Cell number regulator 8	CNR8
14	MAIZE	A0A096PWM1_MAIZE	564	5.80E-21	76.4	PLAC8 family protein	Zm00001d028809
14	MAIZE	K7U2J0_MAIZE	445	9.30E-19	69.4	PLAC8 family protein	Zm00001d052297
14	MAIZE	A0A1D6N835_MAIZE	540	9.40E-19	69.4	PLAC8 family protein	100501228
14	MAIZE	B4G0W3_MAIZE	565	3.60E-18	67.5	PLAC8 family protein	100274421
14	MAIZE	C0P6U5_MAIZE	499	2.50E-17	64.8	PLAC8 family protein	100382355
14	MAIZE	A0A1D6ML42_MAIZE	255	3.60E-17	64.3	PLAC8 family protein	Zm00001d039776
14	MAIZE	A0A1D6M2T4_MAIZE	523	3.80E-17	64.2	SAG20	Zm00001d038030
14	MAIZE	A0A1D6IER6_MAIZE	304	6.60E-17	63.4	PLAC8 family protein	100194349
14	MAIZE	B4FTT4_MAIZE	571	4.10E-16	60.9	PLAC8 family protein	100273234
14	MAIZE	A0A1D6JP06_MAIZE	417	4.50E-16	60.7	Cell number regulator 13	Zm00001d027722
14	MAIZE	CNR13_MAIZE	428	5.90E-16	60.4	Cell number regulator 13	CNR13
15	SORBI	C5WU02_SORBI	168	2.60E-29	103	Uncharacterized protein	SORBI_3001G024700
15	SORBI	C5XVR6_SORBI	181	2.20E-28	100	Uncharacterized protein	SORBI_3004G192100
15	SORBI	C5Y9V9_SORBI	154	2.80E-28	99.7	Uncharacterized protein	SORBI_3006G107900
15	SORBI	A0A1Z5S4D8_SORBI	148	3.00E-27	96.4	Uncharacterized protein	SORBI_3001G024800
15	SORBI	C5WU00_SORBI	173	2.60E-26	93.4	Uncharacterized protein	SORBI_3001G024500
15	SORBI	A0A1Z5RLN0_SORBI	123	1.50E-25	90.9	Uncharacterized protein	SORBI_3004G041000
15	SORBI	C5XGK2_SORBI	183	3.00E-25	90	Uncharacterized protein	SORBI_3003G125100
15	SORBI	C5XST8_SORBI	188	2.80E-24	86.9	Uncharacterized protein	SORBI_3004G308100
15	SORBI	A0A1B6QR78_SORBI	250	7.20E-23	82.4	Uncharacterized protein	SORBI_3001G542900
15	SORBI	C5XP89_SORBI	236	1.90E-21	77.8	Uncharacterized protein	SORBI_3003G063200
15	SORBI	C5WNE8_SORBI	574	7.20E-20	72.7	Uncharacterized protein	SORBI_3001G401800
15	SORBI	C5XAY8_SORBI	276	7.90E-19	69.4	Uncharacterized protein	SORBI_3002G335300
15	SORBI	C5XNW3_SORBI	513	1.00E-18	69	Uncharacterized protein	SORBI_3003G340900
15	SORBI	A0A1B6PAX9_SORBI	493	1.40E-18	68.6	Uncharacterized protein	SORBI_3008G009100
15	SORBI	C5YW73_SORBI	510	3.90E-18	67.2	Uncharacterized protein	SORBI_3009G100100
15	SORBI	C5YZQ7_SORBI	566	3.90E-18	67.2	Uncharacterized protein	SORBI_3009G171700
15	SORBI	A0A1Z5R442_SORBI	466	2.80E-17	64.4	Uncharacterized protein	SORBI_3008G001800

taxon_no	taxon_id	target_name	length	E-value	E_score	target_description	gene_name
15	SORBI	C5WXT4_SORBI	420	8.90E-16	59.6	Uncharacterized protein	SORBI_3001G499200
15	SORBI	A0A1B6PAI4_SORBI	623	2.60E-15	58.1	Uncharacterized protein	SORBI_3008G009200
16	AQUCA	A0A2G5DS22_AQUCA	159	4E-27	95.6	Uncharacterized protein	AQUCO_01500087v1
16	AQUCA	A0A2G5F6D0_AQUCA	199	8.4E-27	94.5	Uncharacterized protein	AQUCO_00201131v1
16	AQUCA	A0A2G5CWD0_AQUCA	238	4.7E-24	85.7	Uncharacterized protein	AQUCO_03500149v1
16	AQUCA	A0A2G5DPL7_AQUCA	140	2.1E-23	83.6	Uncharacterized protein	AQUCO_01700773v1
16	AQUCA	A0A2G5D839_AQUCA	320	2.1E-23	83.6	Uncharacterized protein	AQUCO_02600254v1
16	AQUCA	A0A2G5DPL5_AQUCA	134	3.3E-23	83	Uncharacterized protein	AQUCO_01700774v1
16	AQUCA	A0A2G5F6G1_AQUCA	152	2.6E-21	76.9	Uncharacterized protein	AQUCO_00201130v1
16	AQUCA	A0A2G5D515_AQUCA	251	6.9E-20	72.3	Uncharacterized protein	AQUCO_02700079v1
16	AQUCA	A0A2G5EQS4_AQUCA	582	3.6E-18	66.8	Uncharacterized protein	AQUCO_00500199v1
16	AQUCA	A0A2G5C987_AQUCA	429	3.6E-16	60.4	Uncharacterized protein	AQUCO_07500029v1
17	VITVI	F6HFF3_VITVI	276	3.30E-45	153.8	Uncharacterized protein	VIT_01s0011g05420
17	VITVI	D7T8N4_VITVI	223	9.90E-29	101	Uncharacterized protein	VIT_01s0011g05450
17	VITVI	F6HFF2_VITVI	180	7.90E-28	98.1	Uncharacterized protein	VIT_01s0011g05430
17	VITVI	F6HFF1_VITVI	180	6.80E-27	95.1	Uncharacterized protein	VIT_01s0011g05440
17	VITVI	D7T8N2_VITVI	201	1.00E-26	94.5	Uncharacterized protein	VIT_01s0011g05470
17	VITVI	F6HQB9_VITVI	181	6.50E-26	91.9	Uncharacterized protein	VIT_03s0063g00570
17	VITVI	D7T975_VITVI	209	8.10E-26	91.6	Uncharacterized protein	VIT_01s0011g03260
17	VITVI	D7T977_VITVI	170	2.40E-25	90.1	Uncharacterized protein	VIT_01s0011g03240
17	VITVI	D7SIX0_VITVI	313	7.30E-25	88.6	Uncharacterized protein	VIT_17s0000g03960
17	VITVI	D7TPJ8_VITVI	216	8.90E-25	88.3	Uncharacterized protein	VIT_03s0063g00550
17	VITVI	D7TPJ9_VITVI	217	3.50E-24	86.4	Uncharacterized protein	VIT_03s0063g00560
17	VITVI	D7TT47_VITVI	315	3.70E-24	86.3	Uncharacterized protein	VIT_14s0006g03190
17	VITVI	D7U0G9_VITVI	193	5.30E-24	85.8	Uncharacterized protein	VIT_09s0002g04180
17	VITVI	D7T1Z5_VITVI	239	4.30E-23	82.9	Uncharacterized protein	VIT_16s0022g00450
17	VITVI	D7SLS5_VITVI	277	1.30E-21	78.2	Uncharacterized protein	VIT_15s0021g02460
17	VITVI	F6HR06_VITVI	240	1.90E-19	71.2	Uncharacterized protein	VIT_08s0040g02230
17	VITVI	D7UDQ8_VITVI	249	2.30E-19	70.9	Uncharacterized protein	VIT_00s0203g00180
17	VITVI	F6HC94_VITVI	711	4.60E-17	63.6	Uncharacterized protein	VIT_13s0067g02190
17	VITVI	F6H5I8_VITVI	420	4.80E-15	57.1	Uncharacterized protein	VIT_00s0349g00020
17	VITVI	F6HQT7_VITVI	141	8.40E-15	56.3	Uncharacterized protein	VIT_08s0040g00140
18	POPTR	U5G1J9_POPTR	193	3.10E-28	99.9	Uncharacterized protein	POPTR_010G109100v3
18	POPTR	A0A3N7FME4_POPTR	214	1.90E-27	97.4	Uncharacterized protein	POPTR_010G108900
18	POPTR	B9HJE4_POPTR	192	2.00E-26	94.1	Uncharacterized protein	POPTR_008G132900v3
18	POPTR	A0A2K1ZGI5_POPTR	192	3.20E-26	93.4	Uncharacterized protein	POPTR_008G132800v3
18	POPTR	B9HWK3_POPTR	200	4.70E-26	92.9	Uncharacterized protein	POPTR_010G127700v3
18	POPTR	A0A3N7HIE7_POPTR	181	3.00E-25	90.3	Uncharacterized protein	POPTR_012G092200
18	POPTR	B9HW36_POPTR	193	5.00E-25	89.6	Uncharacterized protein	POPTR_010G108800v3
18	POPTR	A0A2K1ZP42_POPTR	233	1.20E-24	88.3	Uncharacterized protein	POPTR_007G042800v3
18	POPTR	A0A2K1ZVQ5_POPTR	178	1.50E-24	88	Uncharacterized protein	POPTR_006G024900v3
18	POPTR	B9HWK5_POPTR	157	3.60E-24	86.8	Uncharacterized protein	POPTR_010G127800v3
18	POPTR	B9GVE4_POPTR	234	4.30E-23	83.4	Uncharacterized protein	POPTR_003G108800v3
18	POPTR	B9GLH8_POPTR	234	7.40E-23	82.6	Uncharacterized protein	POPTR_001G124500v3
18	POPTR	A0A3N7FWI6_POPTR	181	2.10E-22	81.2	Uncharacterized protein	POPTR_012G060951
18	POPTR	B9GPA2_POPTR	247	5.30E-21	76.7	Uncharacterized protein	POPTR_002G141600v3
18	POPTR	A0A2K1YFA4_POPTR	259	5.70E-20	73.4	Uncharacterized protein	POPTR_011G040100v3
18	POPTR	A0A2K1R581_POPTR	259	6.50E-20	73.2	Uncharacterized protein	POPTR_T144700v3
18	POPTR	A0A2K1XF09_POPTR	586	1.20E-19	72.4	Uncharacterized protein	POPTR_016G129700v3
18	POPTR	A0A2K1R575_POPTR	261	4.10E-19	70.6	Uncharacterized protein	POPTR_T145200v3
18	POPTR	A0A2K2ANY8_POPTR	261	6.90E-19	69.9	Uncharacterized protein	POPTR_004G026700v3
18	POPTR	B9MT99_POPTR	248	1.10E-18	69.3	Uncharacterized protein	POPTR_006G130300v3
18	POPTR	A0A3N7FNE4_POPTR	556	3.10E-18	67.8	Uncharacterized protein	POPTR_010G182100
18	POPTR	A9P9B1_POPTR	241	2.00E-17	65.2	Uncharacterized protein	POPTR_016G087200v3
18	POPTR	U5G1Z7_POPTR	539	3.70E-16	61.1	Uncharacterized protein	POPTR_008G075300v3
18	POPTR	A0A2K1YWA1_POPTR	388	1.50E-15	59.2	Uncharacterized protein	POPTR_010G184700v3
18	POPTR	U5GEX3_POPTR	419	1.20E-14	56.3	Uncharacterized protein	POPTR_005G110000v3
18	POPTR	A0A3N7FRT3_POPTR	63	4.20E-06	28.9	Uncharacterized protein	POPTR_010G109001
19	MEDTR	G7LAH7_MEDTR	191	2.50E-28	100.4	Plant cadmium resistance protein	11407582
19	MEDTR	G7LAH9_MEDTR	169	2.20E-27	97.4	Plant cadmium resistance protein	11413694
19	MEDTR	G7K803_MEDTR	193	4.80E-26	93.1	Plant cadmium resistance protein	11408586
19	MEDTR	G7JLA3_MEDTR	197	1.90E-25	91.2	Fruit weight 2.2 protein	11444523
19	MEDTR	A0A072UD93_MEDTR	252	3.40E-25	90.4	Plant cadmium resistance protein	11427455
19	MEDTR	G7I4T3_MEDTR	137	1.10E-24	88.7	PLAC8 family protein	11430530
19	MEDTR	G7JLA2_MEDTR	161	1.20E-24	88.6	Fruit weight 2.2 protein	MTR_4g086320
19	MEDTR	Q2HU50_MEDTR	171	1.60E-24	88.2	Plant cadmium resistance protein	11431445
19	MEDTR	A0A072UU43_MEDTR	237	1.70E-22	81.7	Cell number regulator-like protein	25490345
19	MEDTR	G7KT54_MEDTR	240	2.80E-22	81	Cell number regulator-like protein	11437234
19	MEDTR	A0A072THP7_MEDTR	244	6.80E-22	79.8	Cell number regulator-like protein	25480545
19	MEDTR	A0A072UMH2_MEDTR	149	1.50E-21	78.7	PLAC8 family protein	25496874
19	MEDTR	A0A072V3T4_MEDTR	247	3.60E-20	74.2	PLAC8 family protein	25485903
19	MEDTR	G7LAH4_MEDTR	147	1.00E-19	72.8	Plant cadmium resistance protein	11406030
19	MEDTR	G7J2F9_MEDTR	498	2.70E-19	71.4	PLAC8 family protein	11406160
19	MEDTR	G7J2F8_MEDTR	512	3.20E-19	71.2	PLAC8 family protein	11413809
19	MEDTR	Q2HTU7_MEDTR	253	4.90E-19	70.6	PLAC8 family protein	11439084
19	MEDTR	G7K7B5_MEDTR	561	8.90E-19	69.8	PLAC8 family protein	MTR_5g083740
19	MEDTR	G7L1S3_MEDTR	231	1.10E-18	69.5	Cell number regulator-like protein	11434752
19	MEDTR	G7JXK8_MEDTR	416	2.10E-15	58.9	MID1-complementing activity-like protein	11421841
19	MEDTR	G7LB65_MEDTR	424	9.70E-14	53.6	MID1-complementing activity-like protein	11445352
19	MEDTR	G7KBH8_MEDTR	132	5.70E-09	38.3	PLAC8 family protein	MTR_5g065810
20	CUCSA	A0A0A0KIS6_CUCSA	150	1.80E-27	96.6	Uncharacterized protein	Csa_5G027940
20	CUCSA	A0A0A0L6I3_CUCSA	199	9.20E-24	84.7	Uncharacterized protein	Csa_3G099630
20	CUCSA	A0A0A0L8B8_CUCSA	239	1.90E-23	83.7	Uncharacterized protein	Csa_3G175650
20	CUCSA	A0A0A0KDV6_CUCSA	236	2.80E-22	79.9	Uncharacterized protein	Csa_6G108550
20	CUCSA	A0A0A0KN60_CUCSA	250	4.10E-19	69.8	Uncharacterized protein	Csa_6G516790
20	CUCSA	A0A0A0K7T1_CUCSA	553	8.40E-19	68.8	Uncharacterized protein	Csa_6G013870
20	CUCSA	A0A0A0KY50_CUCSA	506	1.90E-18	67.7	Uncharacterized protein	Csa_4G325530
20	CUCSA	A0A0A0KH41_CUCSA	254	3.10E-17	63.8	Uncharacterized protein	Csa_6G444990
20	CUCSA	A0A0A0L3N0_CUCSA	418	6.80E-16	59.5	Uncharacterized protein	Csa_3G002640
21	GOSRA	A0A0D2QXW2_GOSRA	145	7.80E-29	101.7	Uncharacterized protein	B456_002G175300
21	GOSRA	A0A0D2NFY6_GOSRA	182	2.50E-28	100	Uncharacterized protein	B456_002G174700
21	GOSRA	A0A0D2M4S3_GOSRA	131	2.70E-27	96.7	Uncharacterized protein	B456_002G175100
21	GOSRA	A0A0D2NDM3_GOSRA	195	1.60E-26	94.2	Uncharacterized protein	B456_005G159200
21	GOSRA	A0A0D2M4S0_GOSRA	185	5.10E-26	92.6	Uncharacterized protein	B456_002G174600
21	GOSRA	A0A0D2Q5R8_GOSRA	182	1.00E-25	91.6	Uncharacterized protein	B456_002G174500
21	GOSRA	A0A0D2RXY8_GOSRA	154	1.40E-25	91.2	Uncharacterized protein	B456_006G062300
21	GOSRA	A0A0D2V937_GOSRA	171	3.10E-25	90.1	Uncharacterized protein	B456_013G043200
21	GOSRA	A0A0D2VU55_GOSRA	193	4.50E-25	89.6	Uncharacterized protein	B456_012G012300
21	GOSRA	A0A0D2S3G8_GOSRA	199	5.10E-25	89.4	Uncharacterized protein	B456_012G150200
21	GOSRA	A0A0D2S6C5_GOSRA	268	6.30E-25	89.1	Uncharacterized protein	B456_006G195300
21	GOSRA	A0A0D2Q5S5_GOSRA	184	4.00E-24	86.5	Uncharacterized protein	B456_002G175400
21	GOSRA	A0A0D2NFZ3_GOSRA	123	1.60E-23	84.6	Uncharacterized protein	B456_002G175600
21	GOSRA	A0A0D2T6X6_GOSRA	173	1.70E-23	84.5	Uncharacterized protein	B456_008G184800
21	GOSRA	A0A0D2QGB0_GOSRA	137	2.00E-23	84.3	Uncharacterized protein	B456_006G252400
21	GOSRA	A0A0D2T6V2_GOSRA	341	1.10E-22	82	Uncharacterized protein	B456_007G006900
21	GOSRA	A0A0D2QIX7_GOSRA	235	4.20E-22	80	Uncharacterized protein	B456_003G113300
21	GOSRA	A0A0D2SGP9_GOSRA	245	6.20E-22	79.5	Uncharacterized protein	B456_007G087500
21	GOSRA	A0A0D2QYU3_GOSRA	255	8.00E-21	75.9	Uncharacterized protein	B456_004G039800
21	GOSRA	A0A0D2SVP0_GOSRA	586	1.20E-19	72.2	Uncharacterized protein	B456_010G192900
21	GOSRA	A0A0D2U5G4_GOSRA	239	2.10E-19	71.4	Uncharacterized protein	B456_008G184700
21	GOSRA	A0A0D2QX05_GOSRA	240	1.20E-18	69	Uncharacterized protein	B456_009G419400
21	GOSRA	A0A0D2QIA2_GOSRA	248	4.00E-18	67.3	Uncharacterized protein	B456_002G249400
21	GOSRA	A0A0D2MHL8_GOSRA	418	1.50E-15	59	Uncharacterized protein	B456_003G007400
21	GOSRA	A0A0D2UNV6_GOSRA	253	2.20E-15	58.5	Uncharacterized protein	B456_009G174900

taxon_no	taxon_id	target_name	length	E-value	E_score	target_description	gene_name
21	GOSRA	A0A0D2VH53_GOSRA	245	2.30E-15	58.4	Uncharacterized protein	B456_011G021200
21	GOSRA	A0A0D2THW9_GOSRA	416	4.20E-15	57.6	Uncharacterized protein	B456_007G192000
21	GOSRA	A0A0D2QM64_GOSRA	136	1.60E-10	42.9	Uncharacterized protein	B456_003G023000
21	GOSRA	A0A0D2R5J9_GOSRA	105	1.60E-06	30	Uncharacterized protein	B456_007G374200
22	BRAOL	A0A0D3DX90_BRAOL	151	5.80E-29	102.6	Uncharacterized protein	106312399
22	BRAOL	A0A0D3DX95_BRAOL	184	8.90E-29	102.1	Uncharacterized protein	Bo8g106450
22	BRAOL	A0A0D3DX96_BRAOL	151	4.20E-28	99.9	Uncharacterized protein	106308080
22	BRAOL	A0A0D3CZ10_BRAOL	163	7.60E-27	95.9	Uncharacterized protein	106299520
22	BRAOL	A0A0D3DX94_BRAOL	147	9.40E-27	95.6	Uncharacterized protein	106312400
22	BRAOL	A0A0D3CAB3_BRAOL	151	3.00E-26	93.9	Uncharacterized protein	Bo5g020270
22	BRAOL	A0A0D3DX89_BRAOL	151	8.20E-26	92.5	Uncharacterized protein	106312398
22	BRAOL	A0A0D3CIV8_BRAOL	133	1.20E-25	92.1	Uncharacterized protein	106294270
22	BRAOL	A0A0D3BP01_BRAOL	187	3.00E-25	90.7	Uncharacterized protein	106342585
22	BRAOL	A0A0D3E5L2_BRAOL	150	3.90E-25	90.4	Uncharacterized protein	106317115
22	BRAOL	A0A0D3AA38_BRAOL	145	1.30E-24	88.7	Uncharacterized protein	106301967
22	BRAOL	A0A0D2ZUN1_BRAOL	155	3.10E-24	87.5	Uncharacterized protein	Bo01320s050
22	BRAOL	A0A0D3CZ12_BRAOL	161	1.10E-23	85.7	Uncharacterized protein	106298690
22	BRAOL	A0A0D3CQ69_BRAOL	188	1.10E-23	85.7	Uncharacterized protein	106299289
22	BRAOL	A0A0D3CPE5_BRAOL	175	5.90E-23	83.4	Uncharacterized protein	Bo6g018020
22	BRAOL	A0A0D3CXU2_BRAOL	161	7.10E-23	83.1	Uncharacterized protein	106299786
22	BRAOL	A0A0D3CPE7_BRAOL	647	5.50E-22	80.3	Uncharacterized protein	Bo6g018040
22	BRAOL	A0A0D3BPZ9_BRAOL	243	1.60E-21	78.7	Uncharacterized protein	106342317
22	BRAOL	A0A0D3C4Q0_BRAOL	234	6.80E-21	76.8	Uncharacterized protein	106336659
22	BRAOL	A0A0D3B5C5_BRAOL	241	2.60E-20	74.9	Uncharacterized protein	106328823
22	BRAOL	A0A0D3EA13_BRAOL	234	4.30E-20	74.2	Uncharacterized protein	106318730
22	BRAOL	A0A0D3AMZ2_BRAOL	453	7.70E-18	67	Uncharacterized protein	Bo2g050500
22	BRAOL	A0A0D3CLB0_BRAOL	522	3.80E-17	64.8	Uncharacterized protein	106295673
22	BRAOL	A0A0D3EHM3_BRAOL	423	4.80E-17	64.4	Uncharacterized protein	106313461
22	BRAOL	A0A0D3C9A5_BRAOL	247	4.80E-17	64.4	Uncharacterized protein	106292465
22	BRAOL	A0A0D3E374_BRAOL	411	9.40E-16	60.3	Uncharacterized protein	Bo9g024720
22	BRAOL	A0A0D3A1N3_BRAOL	420	1.20E-15	60	Uncharacterized protein	106303054
22	BRAOL	A0A0D3DHX0_BRAOL	412	1.80E-15	59.4	Uncharacterized protein	106301973
22	BRAOL	A0A0D3CFR3_BRAOL	236	3.30E-06	29.7	Uncharacterized protein	Bo5g072940
23	ARATH	PCR3_ARATH	152	9.90E-29	100.9	Protein PLANT CADMIUM RESISTANCE 3	PCR3
23	ARATH	PCR2_ARATH	152	1.20E-28	100.6	Protein PLANT CADMIUM RESISTANCE 2	PCR2
23	ARATH	PCR11_ARATH	160	1.10E-27	97.5	Protein PLANT CADMIUM RESISTANCE 11	PCR11
23	ARATH	PCR7_ARATH	133	3.50E-26	92.7	Protein PLANT CADMIUM RESISTANCE 7	PCR7
23	ARATH	PCR10_ARATH	190	9.90E-26	91.2	Protein PLANT CADMIUM RESISTANCE 10	PCR10
23	ARATH	PCR1_ARATH	151	2.70E-25	89.9	Protein PLANT CADMIUM RESISTANCE 1	PCR1
23	ARATH	PCR12_ARATH	161	3.00E-25	89.7	Protein PLANT CADMIUM RESISTANCE 12	PCR12
23	ARATH	PCR9_ARATH	148	1.00E-24	88	Protein PLANT CADMIUM RESISTANCE 9	PCR9
23	ARATH	PCR8_ARATH	190	1.00E-24	88	Protein PLANT CADMIUM RESISTANCE 8	PCR8
23	ARATH	PCR4_ARATH	184	2.90E-24	86.5	Protein PLANT CADMIUM RESISTANCE 4	PCR4
23	ARATH	PCR6_ARATH	224	1.10E-23	84.7	Protein PLANT CADMIUM RESISTANCE 6	PCR6
23	ARATH	Q9ZQC8_ARATH	242	7.20E-22	78.8	At2g37110	TAIR:locus:2061728
23	ARATH	PCR5_ARATH	184	7.50E-22	78.8	Protein PLANT CADMIUM RESISTANCE 5	PCR5
23	ARATH	Q8L3T0_ARATH	244	7.80E-22	78.7	PLAC8 family protein	TAIR:locus:2055617
23	ARATH	P94032_ARATH	241	1.10E-20	75	Gene1000 protein	gene1000
23	ARATH	Q8W4N1_ARATH	563	7.40E-18	66	PLAC8 family protein	F9F8.20
23	ARATH	Q8LFV5_ARATH	254	9.80E-18	65.6	At1g11380	TAIR:locus:2200081
23	ARATH	Q5XV52_ARATH	526	3.40E-17	63.9	PLAC8 family protein	TAIR:locus:2153544
23	ARATH	MCAC1_ARATH	421	8.10E-16	59.4	Protein MID1-COMPLEMENTING ACTIVITY 1	MCA1
23	ARATH	MCAC2_ARATH	416	2.50E-15	57.9	Protein MID1-COMPLEMENTING ACTIVITY 2	MCA2
24	ERYGU	A0A022PQY6_ERYGU	193	9.20E-28	97.8	Uncharacterized protein	MIMGU_mgv1a023749mg
24	ERYGU	A0A022RKB0_ERYGU	173	8.80E-27	94.6	Uncharacterized protein	MIMGU_mgv1a023101mg
24	ERYGU	A0A022QN19_ERYGU	148	2.30E-26	93.3	Uncharacterized protein	MIMGU_mgv1a024272mg
24	ERYGU	A0A022R6L2_ERYGU	189	6.00E-26	91.9	Uncharacterized protein	MIMGU_mgv1a014450mg
24	ERYGU	A0A022Q166_ERYGU	171	3.70E-25	89.4	Uncharacterized protein	MIMGU_mgv1a014987mg
24	ERYGU	A0A022Q8I7_ERYGU	239	2.60E-23	83.5	Uncharacterized protein	MIMGU_mgv1a012819mg
24	ERYGU	A0A022QTR7_ERYGU	237	1.10E-22	81.5	Uncharacterized protein	MIMGU_mgv1a012866mg
24	ERYGU	A0A022QMQ3_ERYGU	145	1.80E-22	80.8	Uncharacterized protein	MIMGU_mgv1a023267mg
24	ERYGU	A0A022RFJ2_ERYGU	231	4.40E-22	79.5	Uncharacterized protein	MIMGU_mgv1a013056mg
24	ERYGU	A0A022Q2R5_ERYGU	181	1.10E-20	75.1	Uncharacterized protein	MIMGU_mgv1a014692mg
24	ERYGU	A0A022RQP2_ERYGU	252	3.80E-20	73.3	Uncharacterized protein	MIMGU_mgv1a012363mg
24	ERYGU	A0A022QYL9_ERYGU	127	4.30E-20	73.1	Uncharacterized protein	MIMGU_mgv11b015160mg
24	ERYGU	A0A022R562_ERYGU	261	2.10E-19	71	Uncharacterized protein	MIMGU_mgv1a012116mg
24	ERYGU	A0A022RMB7_ERYGU	235	7.00E-19	69.3	Uncharacterized protein	MIMGU_mgv1a012934mg
24	ERYGU	A0A022R6C7_ERYGU	518	2.90E-18	67.3	Uncharacterized protein	MIMGU_mgv1a004615mg
24	ERYGU	A0A022PWB3_ERYGU	557	7.00E-18	66.1	Uncharacterized protein	MIMGU_mgv1a003890mg
24	ERYGU	A0A022QIS1_ERYGU	251	3.80E-16	60.5	Uncharacterized protein	MIMGU_mgv1a020026mg
24	ERYGU	A0A022QT97_ERYGU	413	9.70E-15	56	Uncharacterized protein	MIMGU_mgv1a007258mg
24	ERYGU	A0A022PTL3_ERYGU	404	1.10E-14	55.8	Uncharacterized protein	MIMGU_mgv1a007522mg
24	ERYGU	A0A022QN24_ERYGU	80	1.80E-14	55.1	Uncharacterized protein	MIMGU_mgv1a021437mg
24	ERYGU	A0A022QWR4_ERYGU	95	2.30E-14	54.8	Uncharacterized protein	MIMGU_mgv1a0145282mg
24	ERYGU	A0A022R2H6_ERYGU	97	8.60E-14	52.9	Uncharacterized protein	MIMGU_mgv11b020736mg
24	ERYGU	A0A022QYV6_ERYGU	95	1.10E-12	49.4	Uncharacterized protein	MIMGU_mgv1a022411mg
24	ERYGU	A0A022R0J6_ERYGU	90	6.40E-10	40.5	Uncharacterized protein	MIMGU_mgv1a0145281mg
24	ERYGU	A0A022QN31_ERYGU	67	2.40E-08	35.5	Uncharacterized protein	MIMGU_mgv1a017554mg
24	ERYGU	A0A022QQ82_ERYGU	93	3.80E-08	34.8	Uncharacterized protein	MIMGU_mgv1a019726mg
24	ERYGU	A0A022QVI2_ERYGU	155	6.90E-08	34	Uncharacterized protein	MIMGU_mgv1a023189mg
25	SOLLC	A0A3Q7E7K0_SOLLC	164	2E-30	106.6	Uncharacterized protein	101250247
25	SOLLC	A0A3Q7FZ88_SOLLC	186	2.2E-28	100.1	Uncharacterized protein	101267985
25	SOLLC	Q9LKV7_SOLLC	163	1.2E-26	94.5	Fw2.2	ORFX
25	SOLLC	A0A3Q7J868_SOLLC	149	1.9E-26	93.9	Uncharacterized protein	101260315
25	SOLLC	A0A3Q7IL17_SOLLC	188	1.7E-25	90.8	Uncharacterized protein	101261673
25	SOLLC	A0A3Q7FUN2_SOLLC	414	6.8E-25	88.9	Uncharacterized protein	101265423
25	SOLLC	A0A3Q7GNK1_SOLLC	240	2.5E-24	87	Uncharacterized protein	Solyc05g051690.3
25	SOLLC	A0A3Q7FNL6_SOLLC	239	3.2E-24	86.7	Uncharacterized protein	Solyc03g093200.3
25	SOLLC	A0A3Q7GXC4_SOLLC	179	4.2E-24	86.3	Uncharacterized protein	Solyc06g066590.3
25	SOLLC	A0A3Q7HY37_SOLLC	241	7.5E-23	82.3	Uncharacterized protein	101252323
25	SOLLC	A0A3Q7J581_SOLLC	133	1E-22	81.9	Uncharacterized protein	Solyc12g013570.2
25	SOLLC	A0A3Q7J8M1_SOLLC	239	1.3E-22	81.6	Uncharacterized protein	101266170
25	SOLLC	A0A3Q7HLD7_SOLLC	219	8.8E-22	78.9	Uncharacterized protein	109120846
25	SOLLC	A0A3Q7FWU9_SOLLC	246	9.8E-22	78.7	Uncharacterized protein	Solyc03g120600.3
25	SOLLC	A0A3Q7GSE6_SOLLC	376	4.9E-19	70.1	Uncharacterized protein	Solyc06g048810.3
25	SOLLC	A0A3Q7IFK8_SOLLC	314	2.2E-18	68	Uncharacterized protein	Solyc08g013910.3
25	SOLLC	A0A3Q7IMK6_SOLLC	306	2E-17	64.9	Uncharacterized protein	Solyc10g084260.2
25	SOLLC	A0A3Q7GV25_SOLLC	505	1.1E-16	62.5	Uncharacterized protein	109118695
25	SOLLC	A0A3Q7F6L0_SOLLC	418	4.9E-16	60.5	Uncharacterized protein	101266587
