# Supplementary material for: Mix and match: Patchwork domain evolution of the land plant-specific Ca2+-permeable mechanosensitive channel MCA
Source: PLoS One. 2021 Apr 15;16(4):e0249735. doi: 10.1371/journal.pone.0249735 (PMC8049495; doi:10.1371/journal.pone.0249735)

S9 Appendix. Species tree used for Notung rooting analyses of domain trees.

CHLRE: *Chlamycomonas reinhardtii*, VOLCA: *Volvox carteri* f. *nagariensis*, KLENI: *Klebsormidium nitens*, CHABU: *Chara braunii*, MARPO: *Marchantia polymorpha*, MapoRu: *Marchantia polymorpha* subsp. *ruderalis*, PHYPA: *Physcomitrella patens*, SELML: *Selaginella moellendorffii*, CMI: *Cycas micholitzii*, TBA: *Taxus baccata*, AMBTC: *Amborella trichopoda*, MUSAM: *Musa acuminata* subsp. *malaccensis*, ORYSJ: *Oryza sativa* subsp. *japonica*, MAIZE: *Zea mays*, SORBI: *Sorghum bicolor*, AQUCA: *Aquilegia coerulea*, VITVI: *Vitis vinifera*, POPTR: *Populus trichocarpa*, MEDTR, *Medicago truncatula*, CUCSA: *Cucumis sativus*, GOSRA: *Gossypium raimondii*, BRAOL: *Brassica oleracea* var. *oleracea*, ARATH: *Arabidopsis thaliana*, ERYGU: *Erythranthe guttata*, SOLLC: *Solanum lycopersicum*

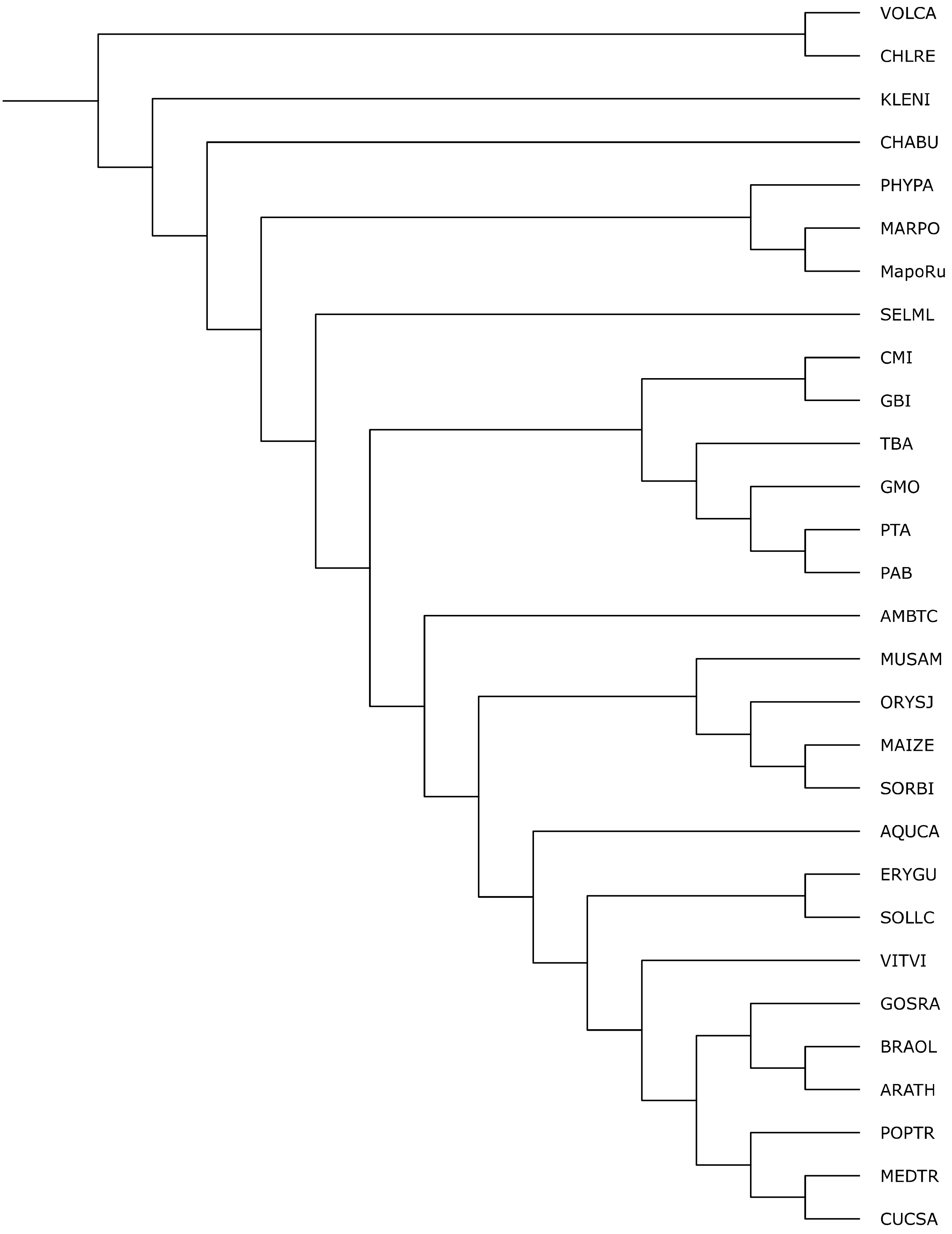

Supplement: S9 Appendix — (PDF) [file pone.0249735.s009.pdf]
