# Supplementary material for: Mix and match: Patchwork domain evolution of the land plant-specific Ca2+-permeable mechanosensitive channel MCA
Source: PLoS One. 2021 Apr 15;16(4):e0249735. doi: 10.1371/journal.pone.0249735 (PMC8049495; doi:10.1371/journal.pone.0249735)

S11 Appendix. MCA^{func} domain ML tree. (*a*) Clade associated with E3 ubiquitin ligase AtPUB13. (*b*) Clade associated with AtPUB45. (*c*) MCA^{func} only proteins. (*d*) Clade associated with AtARO3 and monocot U-box containing protein kinase like proteins. (*e*) MCA clade associated with AtMCA1 and AtMCA2

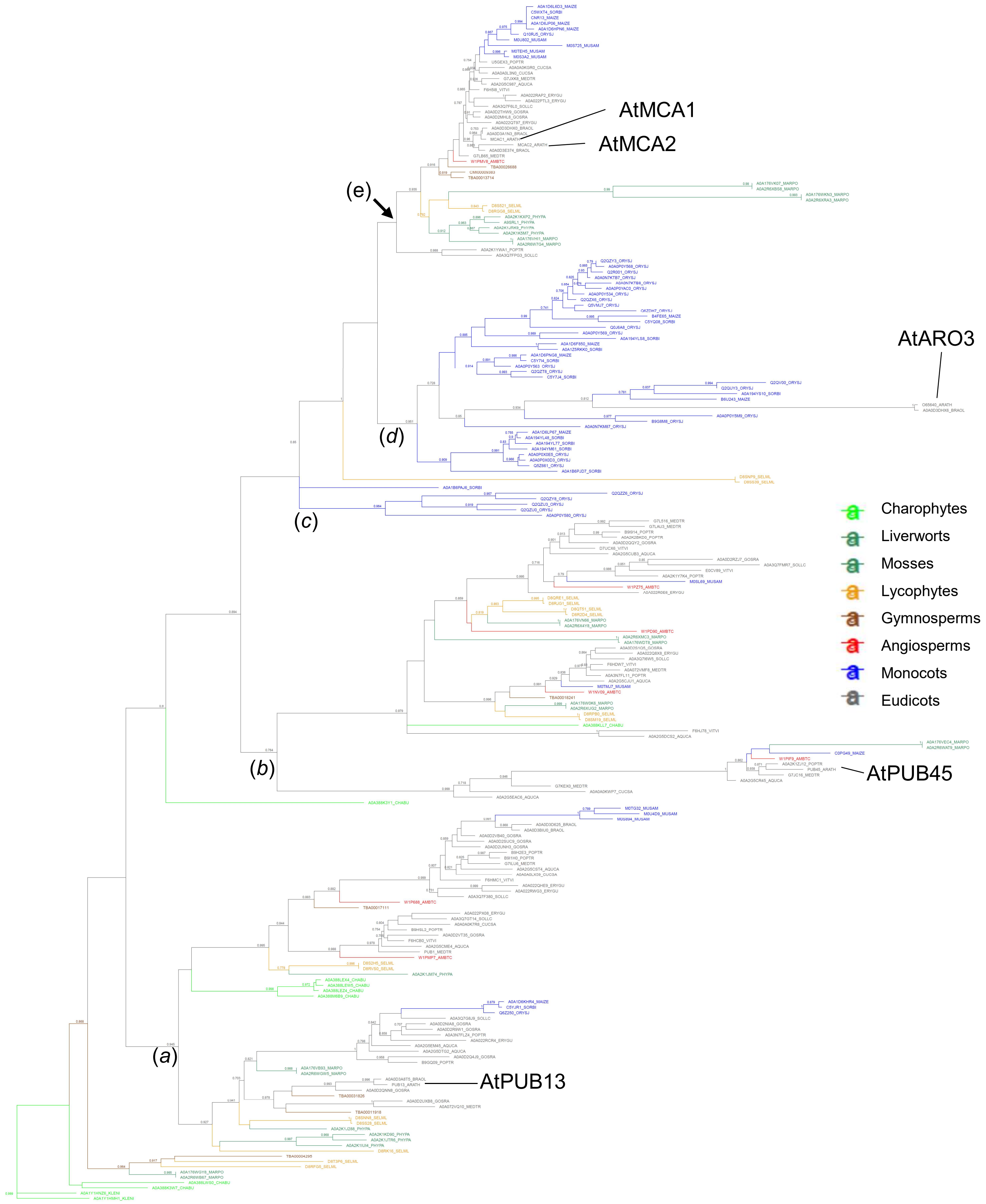

Supplement: S11 Appendix — (a) Clade associated with E3 ubiquitin ligase AtPUB13. (b) Clade associated with AtPUB45. (c) MCAfunc only proteins. (d) Clade associated with AtARO3 and monocot U-box containing protein kinase like proteins. (e) MCA clade associated with AtMCA1 and AtMCA2. (PDF) [file pone.0249735.s011.pdf]
