# Supplementary material for: Mix and match: Patchwork domain evolution of the land plant-specific Ca2+-permeable mechanosensitive channel MCA
Source: PLoS One. 2021 Apr 15;16(4):e0249735. doi: 10.1371/journal.pone.0249735 (PMC8049495; doi:10.1371/journal.pone.0249735)

S12 Appendix. Result of the Notung rooting analysis of the PLAC8 domain tree. Multiple branches show equally strong estimates as possible root positions (marked in red)

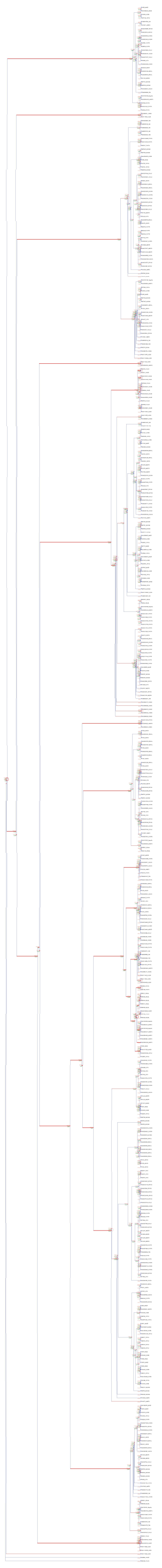

Supplement: S12 Appendix — Multiple branches show equally strong estimates as possible root positions (marked in red). (PDF) [file pone.0249735.s012.pdf]
