# Supplementary material for: Mix and match: Patchwork domain evolution of the land plant-specific Ca2+-permeable mechanosensitive channel MCA
Source: PLoS One. 2021 Apr 15;16(4):e0249735. doi: 10.1371/journal.pone.0249735 (PMC8049495; doi:10.1371/journal.pone.0249735)

S15 Appendix. Species tree used for Notung analyses for full MCA protein sequence tree.

ANTAN: *Anthoceros angustus*, MARPO: *Marchantia polymorpha*, SPHFA*: Sphagnum fallax*, PHYPA: *Physcomitrella patens*, SELML: *Selaginella moellendorffii*, AZOFI: *Azolla filiculoides*, SALCU: *Salvinia cucullata*, CMI: *Cycas micholitzii*, GBI: *Ginkgo biloba*, TBA: *Taxus baccata*, WIMI: *Welwitschia mirabilis*, AMBTC: *Amborella trichopoda*, NYMTH: *Nymphaea thermarum*, CINCA: *Cinnamomum camphora*, MAGGR: *Magnolia grandiflora*, SPIPO: *Spirodela polyrhiza*, ZOSMR: *Zostera marina*, ASPOF: *Asparagus officinalis*, SORBI: *Sorghum bicolor*, PANHA: *Panicum hallii*, SETVI: *Setaria viridis*, ORYSJ: *Oryza sativa*, ANACO: *Ananas comosus*, BRADI: *Brachypodium distachyon*, MUSAC: *Musa acuminata*, AQUCO: *Aquilegia coerulea*, NELNU: *Nelumbo nucifera*, KALFE: *Kalanchoe fedtschenkoi*, VITVI: *Vitis vinifera*, LINUS: *Linum usitatissimum*, RICCO: *Ricinus communis*, MANES: *Manihot esculenta*, SALPU: *Salix purpurea*, POPTR: *Populus trichocarpa*, LOTJA: *Lotus japonicas*, TRIPR: *Trifolium pretense*, MEDTR: *Medicago truncatula*, PRUPE: *Prunus persica*, CUCSA: *Cucumis sativus*, EUCGR: *Eucalyptus grandis*, CITSI: *Citrus sinensis*, GOSRA: *Gossypium raimondii*, CARPA: *Carica papaya*, CAPRU: *Capsella rubella*, ARATH: *Arabidopsis thaliana*, BRAOL: *Brassica oleracea* var. *oleracea*, BRAOC: *Brassica oleracea* var. *capitate*, BETVU: *Beta vulgaris*, AMAHP: *Amaranthus hypochondriacus*, ERYGU: *Erythranthe guttata*, TOBAC: *Nicotiana tabacum*, SOLTU: *Solanum tuberosum*, SOLLC: *Solanum lycopersicum*, DAUCA: *Daucus carota*, HELEN: *Helianthus annuus*

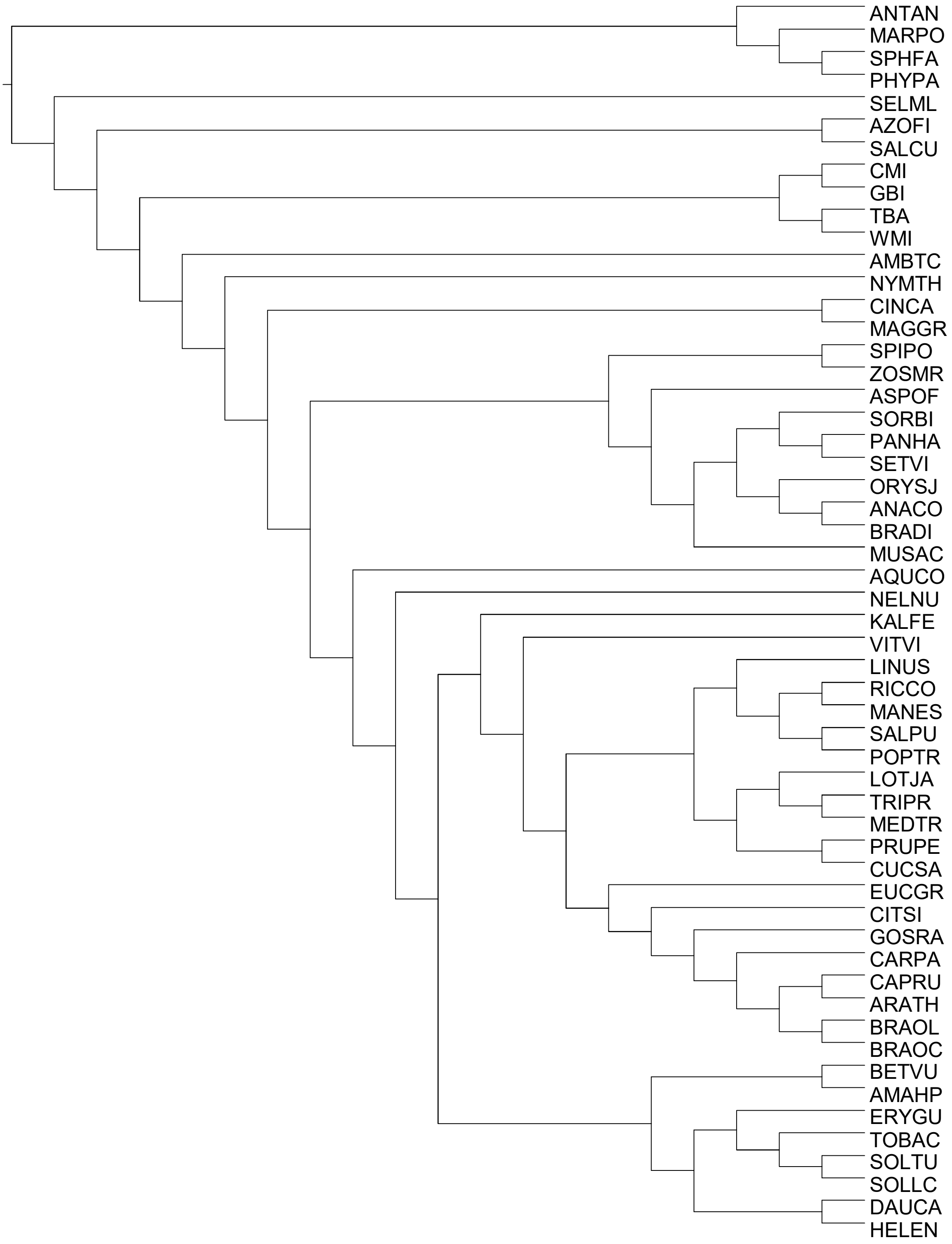

Supplement: S15 Appendix — (PDF) [file pone.0249735.s015.pdf]
