# Supplementary material for: Mix and match: Patchwork domain evolution of the land plant-specific Ca2+-permeable mechanosensitive channel MCA
Source: PLoS One. 2021 Apr 15;16(4):e0249735. doi: 10.1371/journal.pone.0249735 (PMC8049495; doi:10.1371/journal.pone.0249735)

S16 Appendix. Domain partners of MCA[func] domain. Domain partners observed in the MCA[func] domain containing proteins associated with the tree shown in Fig. 1 (left). Domain individual *E* values (i.Evalue) resulting from HMMER website searches are shown as heatmap (right). Absence of domains indicated in grey

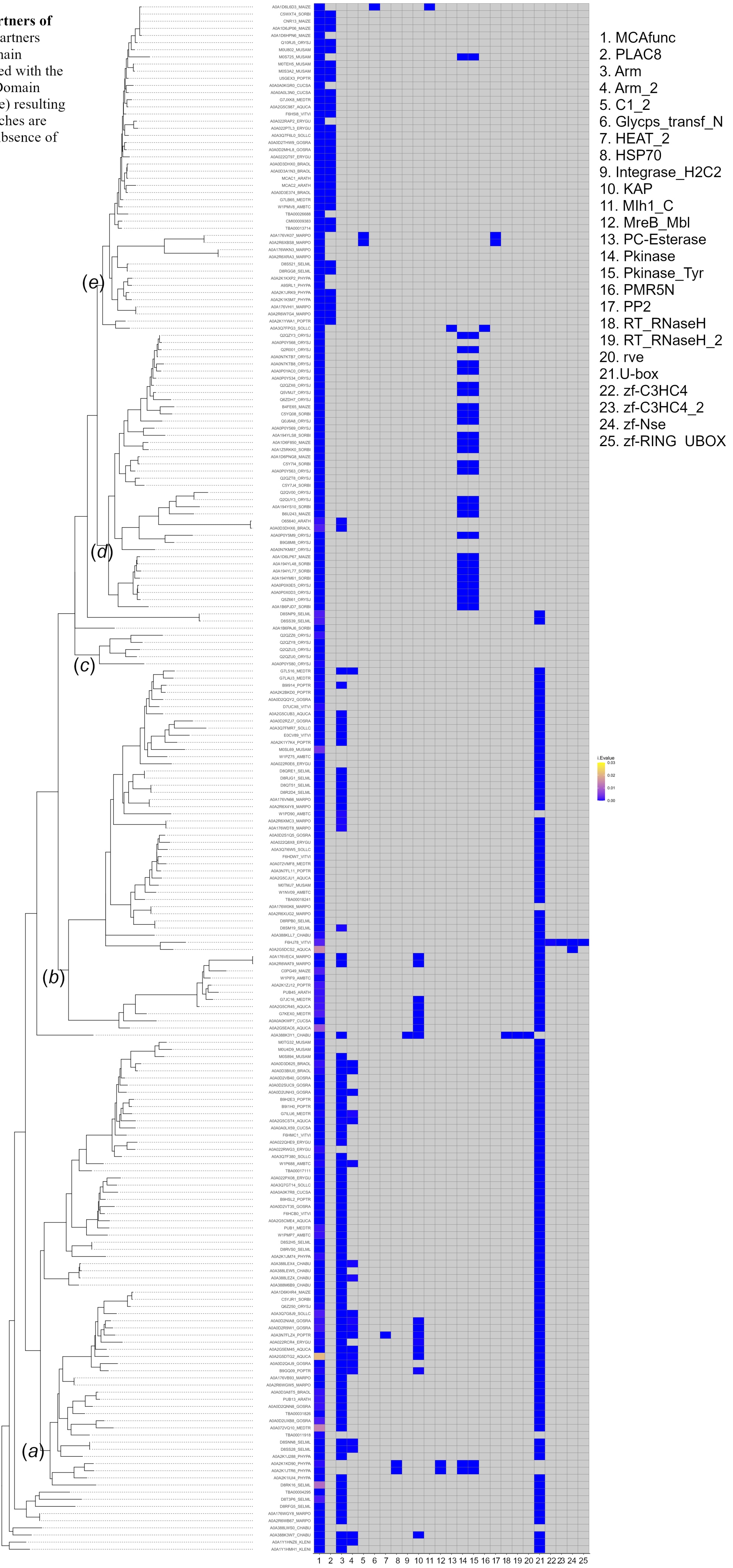

Supplement: S16 Appendix — Domain partners observed in the MCAfunc domain containing proteins associated with the tree shown in Fig 1 (left). Domain individual E values (i.Evalue) resulting from HMMER website searches are shown as heatmap (right). Absence of domains indicated in grey. (PDF) [file pone.0249735.s016.pdf]
