# Supplementary material for: Mix and match: Patchwork domain evolution of the land plant-specific Ca2+-permeable mechanosensitive channel MCA
Source: PLoS One. 2021 Apr 15;16(4):e0249735. doi: 10.1371/journal.pone.0249735 (PMC8049495; doi:10.1371/journal.pone.0249735)

S17 Appendix. Domain partners of PLAC8 domain. Domain partners observed in the PLAC8 domain containing proteins associated with the ML tree (left). Domain individual *E* values (i.Evalue) resulting from HMMER website searches are shown as heatmap (right). Absence of domains indicated in grey

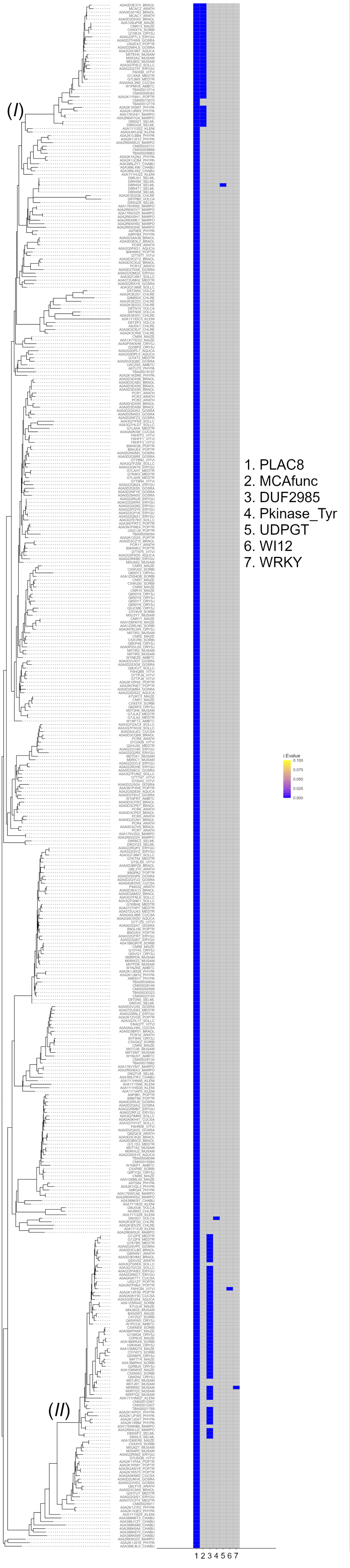

Supplement: S17 Appendix — Domain partners observed in the PLAC8 domain containing proteins associated with the ML tree (left). Domain individual E values (i.Evalue) resulting from HMMER website searches are shown as heatmap (right). Absence of domains indicated in grey. (PDF) [file pone.0249735.s017.pdf]
