# Supplementary material for: Mix and match: Patchwork domain evolution of the land plant-specific Ca2+-permeable mechanosensitive channel MCA
Source: PLoS One. 2021 Apr 15;16(4):e0249735. doi: 10.1371/journal.pone.0249735 (PMC8049495; doi:10.1371/journal.pone.0249735)

S18 Appendix. *MCA* gene duplication events estimated by the Notung analysis. "D" indicates inferred gene duplication event

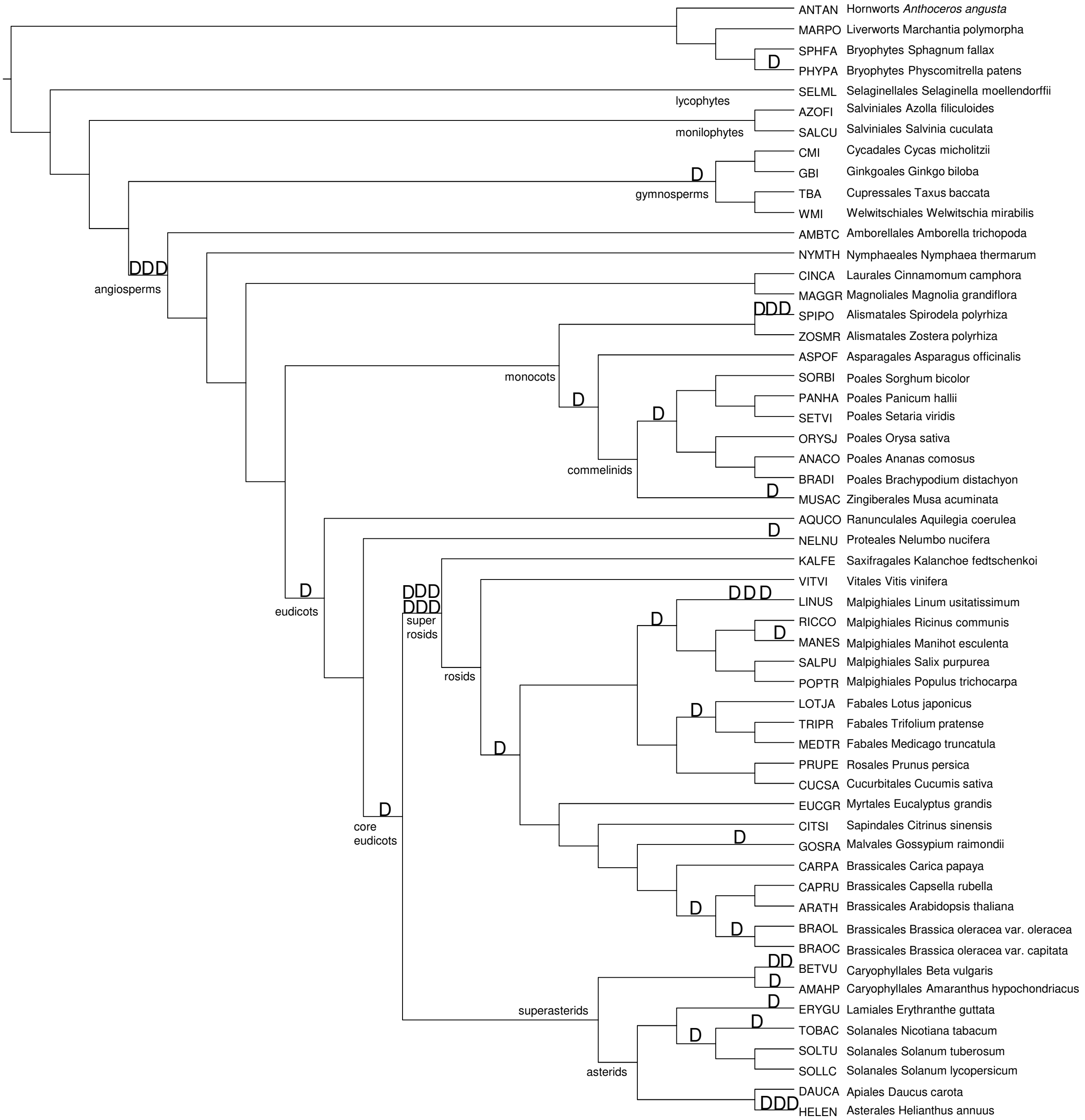

Supplement: S18 Appendix — “D” indicates inferred gene duplication events. (PDF) [file pone.0249735.s018.pdf]
