# Supplementary material for: Mix and match: Patchwork domain evolution of the land plant-specific Ca2+-permeable mechanosensitive channel MCA
Source: PLoS One. 2021 Apr 15;16(4):e0249735. doi: 10.1371/journal.pone.0249735 (PMC8049495; doi:10.1371/journal.pone.0249735)
Supplement: Attachment — Submitted filename: Response_to_Reviewers_MCAr1.docx [file pone.0249735.s020.docx]

PONE-D-20-36297
Mix and match: patchwork domain evolution of the land plant-specific Ca2+-permeable mechanosensitive channel MCA

**Authors` response to the Editor`s general comments**

Thank you very much for the supportive comments from the editor. We greatly appreciate the fact that the manuscript has received such a positive interest in our approach and our findings.

We have revised the manuscript following to the editor and reviewer`s comments as detailed below.

**Editor`s comments**

2. Thank you for stating the following in the Acknowledgments Section of your manuscript:

"RBGE is supported by the Rural and Environment Science and Analytical Services Division (RESAS) in the Scottish Government."

We note that you have provided funding information that is not currently declared in your Funding Statement. However, funding information should not appear in the Acknowledgments section or other areas of your manuscript. We will only publish funding information present in the Funding Statement section of the online submission form.

Please remove any funding-related text from the manuscript and let us know how you would like to update your Funding Statement. Currently, your Funding Statement reads as follows:

"This work was supported by the Japan Society for the Promotion of Science (JSPS) [KAKENHI Grant Number 25120708] to HI, from the Ministry of Education, Culture, Sports, Science & Technology of Japan. KN’s stay at RBGE is financially supported by the Edinburgh Botanic Garden (Sibbald) Trust and the JSPS [JSPS KAKENHI Grant Number 18K06375], and the Sumitomo Foundation."

Please include your amended statements within your cover letter; we will change the online submission form on your behalf.

**Authors` response**

Financial disclosure

"RBGE is supported by the Rural and Environment Science and Analytical Services Division (RESAS) in the Scottish Government."

This sentence is removed from the Acknowledgements and inserted in the Funding Statement section.

The amended Funding Statement is also included in the cover letter.

New Funding Statement is as follows;

"This work was supported by the Japan Society for the Promotion of Science (JSPS) [KAKENHI Grant Number 25120708] to HI, from the Ministry of Education, Culture, Sports, Science & Technology of Japan. KN’s stay at RBGE is financially supported by the Edinburgh Botanic Garden (Sibbald) Trust and the JSPS [JSPS KAKENHI Grant Number 18K06375], and the Sumitomo Foundation. RBGE is supported by the Rural and Environment Science and Analytical Services Division (RESAS) in the Scottish Government."

**Editor`s comment**

3. We note that you have stated that you will provide repository information for your data at acceptance. Should your manuscript be accepted for publication, we will hold it until you provide the relevant accession numbers or DOIs necessary to access your data. If you wish to make changes to your Data Availability statement, please describe these changes in your cover letter and we will update your Data Availability statement to reflect the information you provide.

**Authors` response**

The data submitted to Treebase and protocol.io are now released and available. The Pfam release v.34.0 is under the control of “Pfam” and the “European Bioinformatics Institute” and difficult to arrange individually. Thus we also include the seed alignment of the MCA^func^ domain as supplemental data.

“R codes used in this study are published on protocol.io.　[dx.doi.org/10.17504/protocols.io.bkqwkvxe]

Gene trees and their alignment are available from TreeBASE. [http://purl.org/phylo/treebase/phylows/study/TB2:S26880]

The seed alignment of the MCA^func^ domain will be available in the next release of the Pfam database. [Pfam release v.34.0; PF19584] as well as the S19 Appendix of this study.”

**Editor`s comment**

4. Please upload a new copy of S6 and S17 Figures as the detail is not clear.

**Authors` Comment**

We provided new versions of figures S6 and S17 with higher resolution.

**Reviewer 1**

**Authors` response to the Reviewer 1`s general comments**

We like to thank the reviewer for the very positive comments on our work.

**Reviewer 1`s comment**

1. Major: In many cases the authors refer to a protein or domain evolving in a particular modern group based on their presence in that group as well as in more derived taxa. This is not appropriate without presenting further evidence. The data suggest that the domain or protein most likely arose in the ancestor of all these taxa, which may or may not be a member of the most ancestral group. If the authors know that the common ancestor was a member of the most ancestral modern taxon that evidence must be provided in the manuscript, otherwise the claim must be made only of the common ancestor and not the most ancestral modern taxon. Examples: A) MCAfunc is said to have appeared first in charophytes rather than the ancestor of all streptophytes (lines 29, 303, 348) B) The coupling between MCAfunc and PLAC8 is said to have occurred once, “possibly in the ancestor of bryophytes” (lines 239-242, 348). This should refer to the shared ancestor of all embryophytes. C) several instances in the figure 5 legend. I recommend the authors look for this throughout the paper in case I missed something.

**Authors` response**

Thank you very much for pointing out this evolutionary issue. We agree that MCA^func^ domain might have arisen in the common ancestor of streptophytes, not within charophytes. We also agree that the MCA protein might have originated in the shared ancestor of embryophytes, rather than the ancestor of bryophytes. We have checked the MS and modified all relevant sentences as listed below:

*Lines 28-29 [new lines 28-30]*

*Original text*

“Maximum likelihood (ML) analyses revealed that the MCA^func^ domain first appeared in E3 ubiquitin ligases-like proteins of charophytes.”

*Revised text*

“Maximum likelihood (ML) analyses showed that the MCA^func^ domain observed in E3 ubiquitin ligases-like proteins of charophytes, suggesting the domain arose in the common ancestor of streptophytes.”

*Lines 301-303 [new Lines 332-334]*

*Original text*

“The MCA^func^ domain, on the other hand, was streptophyte-specific and not found in chlorophytes, suggesting that the domain originated in charophytes (Fig 5) [4].”

*Revised text*

“The MCA^func^ domain, on the other hand, was streptophyte-specific and not found in chlorophytes, suggesting that the domain originated in the common ancestor of streptophytes, *i.e.* charophytes plus embryophytes (Fig 6) [4].”

*Lines 322-323 [new Lines 354-355]*

*Original text*

“A key event for the MCA evolution seemed to be the partnering of MCA^func^ and PLAC8 first recruited in bryophytes.”

*Revised text*

“A key event for the MCA evolution seemed to be the partnering of MCA^func^ and PLAC8 first recruited in the common ancestor of embryophytes.”

*Lines 347-348 [new Lines 381-383]*

*Original text*

“In conclusion, MCA is an example of a multidomain protein, whose MCA^func^ domain emerged de novo in charophytes, and recruited an existing domain PLAC8 in bryophytes.”

*Revised text*

“In conclusion, MCA is an example of a multidomain protein, whose MCA^func^ domain emerged de novo in the common ancestor of streptophytes, and recruited an existing domain PLAC8 in the common ancestor of embryophytes.”

*Lines 239-242 [new Lines 247-250]*

*Original text*

“The ML tree topology and distribution of partner domains suggested that the coupling between MCA^func^ and PLAC8 domains occurred once in the plant lineage, possibly in the ancestor of bryophytes, and was sometimes lost after gene duplication events but was always retained in at least one copy.”

*Revised text*

“The ML tree topology and distribution of partner domains suggested that the coupling between MCA^func^ and PLAC8 domains occurred once in the plant lineage, possibly in the common ancestor of embryophytes, and was sometimes lost after gene duplication events but was always retained in at least one copy.”

*Line 348*

See above

*Figure 5 legend*

*Original text*

“**Fig 5. Schematic illustration of MCA evolution.** PLAC8 domain genes are observed from chlorophytes to angiosperms, and many are single domain proteins (a). In charophytes, type I, type II, and type III domain combinations evolved (b). In the ancestor of land plants, from bryophytes onward, type IV domain combinations (MCA type, i.e. MCA^func^ + PLAC8) evolved (c). In addition to the commonly observed types I-IV, lineage specific combinations were also observed.”

*Revised text* [former Fig. 5 is now Fig. 6, new lines 336-341]

“**Fig 6. Schematic illustration of MCA evolution.** PLAC8 domain genes were observed from chlorophytes to angiosperms, and many were single domain proteins (*a*). In the common ancestor of streptophytes, type I, type II, and type III domain combinations evolved (*b*). In the common ancestor of land plants, *i.e.* embryophytes, type IV domain combinations (MCA type, i.e. MCA^func^ + PLAC8) evolved (*c*). In addition to the commonly observed types I-IV, lineage specific combinations were also observed.”

**Minor comments:**

**Reviewer 1`s minor comments**

1. Minor: Some minor grammatical issues were noticed, not enough to be distracting. The authors should edit the full text to identify and resolve as many of these as possible.

**Authors` response**

We revised the manuscript and made the appropriate corrections.

**Reviewer 1`s minor comments**

2. Minor: The groups “streptophytes” and “chlorophytes” are used extensively here. These terms can be useful, but are sometimes used to mean different things in different manuscripts. Figure 5 provides a visual explanation, but it would be much more helpful if these terms were defined in the introduction to resolve any potential confusion while readers progress through the paper.

**Authors` response**

We added sentences for the definitions of terminology with references.

*New Lines 87-91*

“Here, for ranks, we followed the definition by Leliaert et al. [19] and NCBI Taxonomy Browser (https://www.ncbi.nlm.nih.gov/guide/taxonomy/), where viridiplantae include green algae (both of chlorophyte and charophyte) and streptophytes, streptophytes include charophyte algae and embryophytes, and embryophytes (also termed as “land plants”) include bryophytes, ferns, gymnosperms and angiosperms.”

**Reviewer 1`s minor comments**

3. Minor: Authors use incorrect language when discussing the patterns identified using HMMs referring to them as “HMMs”, when they should be referred to as either “profile HMMs” or “profiles” in brief, not “HMMs”. I double checked this with the HMMER User’s Guide. This issue was noticed on lines 25/26, and 107-114 but may also be present in other places.

**Authors` response**

Thank you very much for pointing out our error. We changed HMMs to profile HMMs throughout the manuscript and legends of figure and appendix.

**Reviewer 1`s minor comments**

4. Minor: I recommend the authors remove the statement about AtMCA1 being expressed in hamster ovary cells on lines 64-66. The contextual difference between plant roots and hamster ovaries are enormous. I therefore found it distracting and I’m not convinced it added to the story, but if the authors disagree this is not something I would insist on.

**Authors` response**

The sentence is now removed from lines 64-66

**Reviewer 1`s minor comments**

5. Minor: Authors should expand on the acronym “SMS” on line 134

**Authors` response**

The explanation for the acronym SMS has been added in line 134 (new line 139)

“Maximum likelihood (ML) analyses were carried out with PhyML v.3.0 [24] on the ATGC server (www.atgc-montpellier.fr), with Smart Model Selection (SMS) [25].”

**Reviewer 1`s minor comments**

6. Minor: All figures were low resolution and difficult to read, before publication the authors should replace these with higher resolution versions.

**Authors` response**

We provided high resolution images for all figures.

**General comment from reviewer 2**

Reviewer #2: During this study, the authors examine the domain evolutionary history of the multi-domain plant-specific mechanosensitive channel, MCA. They thoroughly examined the evolution and origin of both MCA domains MCA^func^ and PLAC8, via extensive phylogenetic analysis, and determined that the coupling of both domains occurred via a single event. They reveal that the MCA channel has a complex evolutionary history, however show that at least one full copy of the protein is present in streptophytes, suggesting function of the channel is highly important. The findings presented show an impressive example of how domains can combine, resulting in new protein function and opens up avenues for further studies determining the role of MCA proteins in land plant evolution.

Overall, the study is detailed covering a wide range of plant species and performing extensive analysis to track the evolutionary history of MCA domains. The introduction and discussion sections are clearly written, leading the reader through the logic of the study and are accessible to a wide audience. However, the presentation of the results text is not as clearly written or easy to follow and should be amended prior to publication. As it stands, this section is not accessible to a wider audience.

**Authors` response**

Thank you very much for the supportive comment of our work. We revised the manuscript according to the reviewers` and editor`s comments.

**Reviewer 2`s comment 1**

The results section text lacks suitable explanation and does not provide a narrative leading the reader through the figures. For example, the results text starts “In total, 217 proteins possessing…”, without prior explanation (aside from a brief mention in the introduction) as to which species were investigated or the tools by which the search was conducted. In general, this lack of description continues throughout the results text. The authors are recommended to edit the text so that each results section starts by introducing the reasoning behind obtaining the results in the upcoming figure and the brief method by how the results were obtained. The following section should lead from the one before in the text, rather than be stand alone.

**Authors` response**

We added narrative sentences at the beginning of each result section.

The species list are in Tables and appendices, and thus we cited those files in the results. Since this study included a large number of species, we like to do this in a table format in Table 1. The details of tools used in this study were stated in the Materials and Method section but we also added short descriptions as follows.

New Lines 181-182

“To determine the distribution of MCA proteins in viridiplantae, 25 proteomes (see Table 1) were interrogated for domains of the MCA protein, MCA^func^ and PLAC8, with profile HMMs using HMMER.”

New line 206-207

“Since MCA is a multidomain protein, we studied the phylogenetic relationships of the domains MCA^func^ and PLAC8 separately.”

Line 238

“The phylogeny of the other MCA domain, PLAC8, was also examined phylogenetically.”

New lines 261-264

“While the PLAC8 domain commonly existed within the plant, animal, and fungi kingdoms, the MCA^func^ domain was only observed in streptophytes in the plant kingdom. Thus, the MCA^func^ domain might be the key domain for the MCA protein, and we further assessed the coupling of the MCA^func^ domain with its partner domains.”

New lines 291-293

“In order to unravel the history of MCA proteins in plants, a phylogeny of full MCA proteins from 55 embryophyte species was reconstructed. The full MCA proteins include both MCA^func^ and PLAC8 domain sequences.”

**Reviewer2`s comment 2**

Additionally, the presentation of Table 1 as it stands is unclear as the domain contribution types are not mentioned at all until the description of Figure 3. The authors should add a description of these earlier in the text with the presentation of the table, or consider separating this information to a second table which follows Figure 3.

**Authors` response**

Thank you for this suggestion. The old Table 1 is now split into two, Table 1 and Table 2. Table 2 is placed after Fig.3 for the ease of reading.

**Reviewer2`s comment 3**

Finally, Appendix S18 is referred significantly within the text and is useful to look at – the authors could consider including this as a main Figure to avoid the reader having to filter through the Supplementary Information.

**Authors` response**

The S18 Appendix now appears as Fig. 5. The Figure numbers were also amended accordingly.

**Reviewer2`s minor comment**

Additional minor edits/suggestions:

- COMPLEMENTING is spelled incorrectly on line 24 of the abstract

**Authors` response**

Thank you for pointing out this error. Now it is corrected.

**Reviewer2`s minor comment**

- There is an extremely long sentence between lines 62-68 of the Introduction which should be separated into several sentences.

**Authors` response**

The sentence in lines 62-68 (new lines 63 –70) are revised as follows.

“In *A. thaliana*, two paralogous *MCA* genes, *AtMCA1* and *AtMCA2* have been isolated, and their functions examined in great detail. The AtMCA1 protein is involved in touch sensing at the root tip and a hypoosmotic shock-induced increase in the cytosolic free Ca^2+^ concentration [7]. AtMCA2 was reported to participate in Ca^2+^ uptake at the roots [10]. In addition, AtMCA1 and AtMCA2 respond to membrane stretch to generate cation currents when expressed in *Xenopus laevis* oocytes [8]. Furthermore, MCA channels appear to have common functions in plants, based on studies on *Oryza sativa* OsMCA1 [11,12,13], *Nicotiana tabacum* NtMCA1, NtMCA2 [14], *Zea mays* CNR13 [15], and *Streptocarpus MCA*-like gene (as *Saintpaulia* in Ohnishi *et al.* [16]; see Nishii *et al.* [17]).”

**Reviewer2`s minor comment**

- In Line 106 of the Methods the authors should define HMM

**Authors` response**

The sub-header line 106 (new line 112) has been revised according to the reviewer`s comment and “HMM” is spelled out.

**Reviewer2`s minor comment**

- Table 1 legend title is vague and is not stand alone- suggest the authors refine this so it is more descriptive

**Authors` response**

Table 1 is now split to Table 1 and Table 2 following the Reviewer 2`s comment.

The legend of Table 1 has been revised as follows.

**“Table 1. Number of proteins found in proteomes.** Result of profile HMM searches of MCA^func^ and PLAC8 domains in proteomes of 25 taxa across viridiplantae. Number of proteins retaining the MCA^func^ or PLAC8 domains (*E* value < 10^-3^) are listed and arranged following the Tree of Life (see S7, and S8 Appendix).”

**Reviewer2`s minor comment**

- Suggest adding a column with species in Table 1.

**Authors` response**

We are slightly confused by this suggestion, as Table 1 has a “Taxon” column in which the species names are presented.

**Reviewer2`s minor comment**

- In line 200, the authors should define ML

**Authors` response**

We added Maximum Likelihood in line 200 (new line 207) following to the reviewer`s comment.

**Reviewer2`s minor comment**

- Suggest that the authors add the species to the description of Fig3 in lines 253-266. For example K. nitens (charophyte).

**Authors` response**

In lines 253-266 (new lines 261-279), we added the taxonomic groups to the species.

**Reviewer2`s minor comment**

- Appendix S18 is referred to quite a lot within the text and is useful to look at – should this be included as a Figure in its own right to avoid the reader having to filter through the Supplementary Information?

**Authors` response**

Please see above response. S18 Appendix is now as Fig. 5.

**Reviewer2`s minor comment**

- The resolution of S6 Appendix should be improved if possible- it is difficult to see the residue numbers

**Authors` response**

The S6 Appendix is now revised and with higher resolution.

**Reviewer2`s minor comment**

- S9 S15 Appendices- only mnemonics are used. Would recommend providing the full names in the legend as well.

**Authors` response**

The species` full name was added in the legends of S9 and S15 Appendix.

**Reviewer2`s minor comment**

- S16 and S17 Appendices- would suggest the authors list the domain partners on the right of the heatmap as in Figures 1 and 2, rather than at the bottom as they are small and hard to see.

Authors` response

**Authors` response**

We revised S16 and S17 Appendix following to the reviewer`s comment.

**Reviewer #3**

Reviewer #3: In the manuscript “Mix and match: patchwork domain evolution of the land plant-specific Ca2+-permeable mechanosensitive channel MCA” the authors present an investigation of the MCA Ca2+ permeable mechanosensitive ion channel using proteomics and bioinformatics. Their data suggests that the MCA protein is composed of many different portions. This work found that the MCA region of a MCA protein is localized to the N-terminus and is ~170 amino acids.

**Reviewer 3`s comment**

In lines 188-198 you discuss the Table 1 and mention the different classes of the proteomes, can you include the different classes of the genomes in the table?

**Authors` response**

The column “Vernacular name” has been added to Table 1 for the information of taxonomic classes.

**Reviewer 3`s comment**

You utilize a completed and partial proteome for Marchantia polymorpha as two distinct proteomes, while there is a substantial information to be gained from comparing two different experimental conditions. Are the genes identified in the partial proteome the same as the genes identified in the more complete proteome? Unless there are significant differences between the two proteomes can you eliminate the partial from the data set. If there are substantial differences that are not accounted by the completeness of the proteome then please clarify this in the manuscript.

**Authors` response**

Although the proteome completeness of *Marchantia polymorpha* subsp. *ruderalis* is relatively low, adding this gene to the matrix greatly stabilized the resulting trees. Compared to the well-studied angiosperms, there are only very few proteomes available for the basal groups such as bryophyte or lycophytes, but these are important to add. Therefore, we like to keep this proteome information and sequence in this study.

**Reviewer 3`s comment**

-As multiple proteomes are incomplete, can you provide the completeness of the proteomes within the manuscript, perhaps in table 1?

**Authors` response**

The proteome completeness information is added to S2 Appendix. The numbers of proteins registered in each proteomes and their BUSCO completeness were listed. The plaza database of gymnosperm proteomes does not have BUSCO information and thus the information is obtained by the authors. It is added to the materials and methods section.

New lines 107-110

“The proteome completeness information, *i.e.* BUSCO completeness values (BUSCO-C) were available for most taxa on the Uniprot database. The BUSCO-C values of proteomes from plaza database (*Cycas micholitzii*, *Taxus baccata*) were newly obtained in this study using BUSCO v.4.0.6 (Simao et al. 2015), by comparisons against viridiplantae_odb10 lineage datasets.”

**Reviewer 3`s comment**

-You could remove any incomplete proteomes from the analysis as it is difficult to speculate as if a protein is not present in the data set or not present in the wild type cell.

**Authors` response**

The proteomes used here represent the reference proteome for the species. Although many proteomes are not complete (most are between 90-99.8%), they are still regarded as the current reference proteomes data for wild type lineages. The phylogenetic placement of the worst complete genome, the gymnosperm *Cycas micholitzii* (BUSCO-C 36.3%) is consistent with the tree of life and the MCA domain evolution patterns we found. Removal of this and other incomplete proteomes would make the analyses unnecessarily unstable, and we like to keep them included (strictly taken, no proteome is complete, even at BUSCO comparison; see Table S2 Appendix).

**Reviewer 3`s comment**

If the MCA domain is ~420 amino acids (line 71), can you speculate the role of the Type II as they appear to be smaller than the required amount.

**Authors` response**

This is a slightly imprecise comment. To be exact, the MCA protein is approximately 420 amino acids in length and retains two domains (MCA^func^ and PLAC8). The MCA^func^ domain defined in this study is approximately 167 aa length (New line 117).

The Type II domain combination stands for proteins only retaining the MCA^func^ domain (see S7 and S16 Appendix). The function of Type II domain genes is unknown in databases such as Uniprot. It would be too speculative to describe function without functional studies, which are much beyond the scope of our study.

We added the following sentence in the discussion section.

New Lines 368-370

“In addition, there are proteins only retaining the MCA^func^ domain, but their function is not yet reported and remains unknown (S7 Appendix).”

**Reviewer 3`s comment**

In the conclusion can you speculate the role of these proteins and how these are similar or different than the AtMCA1 and AtMCA2 (or other known functional proteins)

**Authors` response**

There is a sentence in the conclusion. We felt that we need to await further studies to be reported to make conclusive statements. We added the following sentences.

New lines 387-392

“In the basal lineage of streptophytes, charophytes, the MCA^func^ domain is associated with U-box and Arm domains, supposed to play roles in the E3 ubiquitin ligase pathway. On the other hand, MCA proteins with MCA^func^ and PLAC8 domains show quite different roles in ion transport. This further supports a hypothesis where domain swapping is an efficient mechanism to increase protein numbers with diversified functions during organismal evolution.”

**Reviewer 3`s comment**

In general many of the supplemental figures and figures are blurry and have made some of the interpretation difficult. Many of them required significant magnification in order to see the content.

**Authors` response**

We revised all main figures and most supplemental figures, and they now have higher resolution and readable text.

Below you can find some specific suggestions

**Reviewer 3`s comment**

Figure 1: the clade designations are difficult to see, can you move them to the left on the figure or highlight the regions using specific colors to make it easier to follow?

**Authors` response**

We added open ellipses to better indicate the clades.

**Reviewer 3`s comment**

Figure 3:

As the genes are arranged by species it is unclear if all of the Type Ib are the same, should they be the same?

**Authors` response**

Type Ib share the same domain composition. They sometimes vary in the length of the sequence regions between the domains.

**Reviewer 3`s comment**

Is there a difference between the two genes from P. patens?

**Authors` response**

This comment is unclear. In P. patens, three, not two, MCA proteins were recognized. These are slightly different in their amino acid sequences. There are two P. patens specific gene types that vary in their Pkinase and Pkinase_Tyr domains at the 3’end (Fig. 3).

**Reviewer 3`s comment**

Are the known MCAs, AtMCA1 and AtMCA2, shown in this graphic?

**Authors` response**

Yes, they are both type IV shown in Fig 3 for *A. thaliana*, having only the MCA^func^ and PLAC8 domains. We added this in Fig. 3 legend.

“The well-studied MCA proteins in *A. thaliana*, AtMCA1 and AtMCA2, are type IV proteins.”
