# Supplementary material for: Mix and match: Patchwork domain evolution of the land plant-specific Ca2+-permeable mechanosensitive channel MCA
Source: PLoS One. 2021 Apr 15;16(4):e0249735. doi: 10.1371/journal.pone.0249735 (PMC8049495; doi:10.1371/journal.pone.0249735)
Supplement: Attachment — Submitted filename: Response_to_Reviewers_r2.docx [file pone.0249735.s021.docx]

**PONE-D-20-36297R1**

**Response to Reviewers**

Editor`s comment

We request to take on the two remaining issues - a re-worded statement in the Abstract, and better-quality figures (they still appear blurry).

**Reviewer`s comment**

Reviewer #1: This manuscript is fundamentally an excellent paper, and it is obvious that the authors have made a strong effort to respond to my comments in the first review response. With two small exceptions I believe it is ready to be accepted for publication at PLOS One. These exceptions are both issues that I mentioned in the first review and will be described in detail below.

1) All figures are still “fuzzy” and difficult to read. I am not sure what the solution is, but I encourage the authors to seek advice and try something new in order to improve their resolution.

2) I identified an issue in my previous review where the evolution of a new domain was attributed to the current most ancestral taxon as opposed to the ancestor of that taxon as well as more derived groups. This was fixed completely in all cases but one. On lines 28-30 in the abstract a claim is still made that the MCAfunc domain first appeared in charophytes, whereas the data suggests it first appeared in the common ancestor of streptophytes. One potential solution would be to change this sentence to, “We identified The MCAfunc domain in all streptophytes including charophytes; based on our Maximum Likelihood (ML) analyses, this suggests the MCAfunc domain evolved early in the history of streptophytes”

Reviewer #2: The authors have suitably addressed all previous comments. Changes to the text and presentations of Tables make for much easier reading.

Reviewer #3: (No Response)

**Authors` response**

We greatly appreciate the positive comments on our manuscript. Our responses to the specific comments are as follows;

**Reviewer 1- Comment 1**

We have contacted the PLOS ONE production team regarding the resolution of the figures and they kindly tested our figures’ quality: the figures were tested through PLOS ONE`s imaging tool (PACE) and all figures passed the review. The email from the production teams is attached below.

We also confirmed that the original TIFF figure files appeared clearly on our screen. We noticed that the generated summary PDF that included all files and figures does not show the figures at the original file quality, and these would not be their final appearance. We are wondering if the reviewer could download (by clicking the box at the top right corner of the figure pages in the pdf file) and review the original TIFF figures, rather than those in the summary PDF.

**Reviewer 1- Comment 2**

We have revised the sentence along the reviewer`s suggestion.

*Lines 28-30*

*Original sentence:*

Maximum likelihood (ML) analyses revealed that the MCA^func^ domain first appeared in E3 ubiquitin ligase-like proteins of charophytes, suggesting the domain arose in the common ancestor of streptophytes.

*Revised sentences:*

We identified the MCA^func^ domain in all streptophytes including charophytes where it appeared in E3 ubiquitin ligase-like proteins. Our Maximum Likelihood (ML) analyses suggested that the MCA^func^ domain evolved early in the history of streptophytes.

**Reviewer 2**

We like to thank reviewer 2.
